# Historical Retrospect for January 1824

**Published:** 1824-01

**Authors:** 


THE LONDON
### Medical and Physical Journal.

1 OF VOL. LI.] JANUARY, 1824. [No. 299.

For many fortunate discoveries in medicine, and for the detection of numerous errors, the world is indebted to the rapid circulation of Monthly Journals; and there never existed any work, to which the Faculty, in Europe and America, were under deeper obligations, than to the Medical and Physical Journal of London, now forming a long, but an invaluable, series.—RUSH.

### HISTORICAL RETROSPECT

### FOR JANUARY 1824.

WE request our readers to observe that the following Paper neither is, nor professes to be, a history of all the medical facts and opinions, whether new or otherwise, that have appeared within the last six months; but merely a compilation of as many of the more important observations, *not already published in this Journal*, as our limits permit us to give, without encroaching too much on the other departments. It is our endeavour to keep pace with the progress of discovery as nearly as we can, by inserting in our "INTELLIGENCE" abbreviated accounts of remarks too extended to be given without curtailment. Notwithstanding this, however, we find, every six months, much matter accumulated on our hands, which we have not been able to lay before our readers. The purpose of the present compilation is to obviate this inconvenience as much as possible; and we therefore proceed to give in succession some of the more important facts connected with medical science, which have come to our knowledge during the last half year.

### NATURAL ANATOMY AND PHYSIOLOGY.

The anatomy of the *brain* has occupied the attention, and been elucidated by the labours of, TIEDEMAN, the celebrated professor at Heidelberg, in a work which has been recently translated into French by M. JOURDAN,* and from which we subjoin a few facts with respect to the development of this organ. The observations of Tiedeman confirm the general principle, that the encephalon is produced by the gradual development of the cranial extremity of the spinal marrow. This, which at first consists of a limpid fluid, gradually becomes opaque,

* *Anatomie du Cerveau, contenant l'Histoire de son Developpement dans le Fœtus, avec une Exposition comparative de sa Structure dans les Animaux.* Par F. TIEDEMAN, &c. Traduite de l'Allemand, avec un Discours preliminaire sur l'Etude de la Physiologie en general, et sur celle du Cerveau en particulier. Par A. I. L. JOURDAN, D.M.P. &c. &c.—Paris, 1823.

pulpy, and at length medullary. The sooner it is examined after the period of conception, the larger it is in proportion to the encephalon. It is shown, with respect to the tubercula quadrigemina, after having followed them through the different epochs of their development, that the two large tubercles situated before the cerebellum in birds, and from which the optic nerves proceed, are neither the optic thalami, as has been supposed, nor the nates, as Gall and Cuvier have thought; but the entire mass of the tubercula quadrigemina, similar to what is observed in man, with this difference alone, that there does not exist between them the transverse separation. The optic thalami and the corpora striata are evidently reflections of the peduncles of the brain; and M. Tiedeman shows, with perspicuity, how these, at first projecting, and not bound down, become gradually covered by the membranes of the hemispheres, and inclosed in the cavity of the lateral ventricles. The observations of this anatomist overturn the system of Dr. Gall with respect to the returning fibres. These fibres, according to Tiedeman, have no real existence, as the parts which they are supposed to form can be discovered before the grey substance from which they are said to be derived. It is thus that the corpus callosum is not formed by fibres of this kind, but by the re-union of the expanded fibres of the peduncles of the brain, which turn upwards and from without inwards, to unite themselves on the median line with those of the opposite side. This body constitutes a commissure between the two hemispheres, which does not appear to be very important, since there are many animals in which it does not exist, and since it has been wanting even in man, without the intellectual faculties being thereby perceptibly disordered.*

The duplicatures of medullary fibres, which constitute the cornu ammonis, have been followed by Tiedeman in all the periods of the development. He shows that the grey or cortical substance, so far from being the matrix of the medullary matter, does not appear till long after it. The grey substance appears to be formed by a multitude of vascular ramifications, and always proves, according to M. Tiedeman, that the parts of the brain where it is found enjoy a very energetic vitality, and a high degree of activity. With respect to the convolutions, this anatomist establishes that they are not, as Gall maintains, produced by the folding of the membrane of the hemispheres, but that the pia mater, turning in upon itself, dips into the soft substance of the brain, and determines their form.

It appears, from some observations of M. Desmoulins, that three states of the encephalon exist in fishes, with regard to the union of the walls of the fourth ventricle and the cerebellum:—1st. Insulated development of the walls of the fourth ventricle, the cerebellum remaining of the ordinary proportions; or insulated development of the cerebellum, the walls of the fourth ventricle remaining of ordinary proportion. In these two cases, the commissure of the fourth ventricle does not adhere to the cerebellum. 2dly. Simultaneous extreme development of the cerebellum, and of the walls of the fourth ventricle, by the union

* *Journal Complémentaire*, Avril 1823.

of two causes, which otherwise produce each of these developments separately,—viz. excess of development of the fifth pair for the cerebellum, and of the eighth pair for the walls of the fourth ventricle. In this case the commissure of the fourth ventricle is united to the cerebellum. 3dly. Excessive development of the walls of the fourth ventricle co-existing with absence of the cerebellum. In this case the commissure of the fourth ventricle adheres to the optic lobes. In frogs, toads, vipers, and adders, the cerebellum is likewise wanting; but the commissure, which does not always exist in the creatures mentioned, does not adhere to the optic lobes in the toad, in which it is, moreover, much smaller than in the frog. In the three combinations above mentioned, there never exists any relation between the pneumogastric nerve and the cerebellum, but constantly between it and the walls of the fourth ventricle.

In the second place, with regard to the composition of the spinal marrow, it appears that, in eight or ten species of fishes, in the tortoise and others, there is not an atom of grey, yellow, or cineritious matter, in the whole length of the spinal cord; so that the opinion which supposed that grey matter always existed in the centre of the spinal marrow, is without foundation; as likewise is that which supposes the white matter to be generated by the grey. M. Desmoulins has likewise ascertained, with Magendie, that, in the spinal marrow of the human embryo, the white matter precedes the grey in the order of its formation, as M. Serres had already observed; so that the grey matter does not proceed from the white, nor the white from the grey, but that each is simultaneously, or successively, formed by the corresponding surface of the pia mater.*

The essays of Reil on the Anatomy of the Brain have been rendered accessible to the English reader by the translation of Mr. Mayo, in a work which likewise contains some interesting original essays on various points of comparative anatomy and physiology.†

A paper of considerable interest, containing various inquiries respecting the *Anatomy of the Eye*, has been published by Dr. Jacob, of Dublin.‡ The first part of his investigation refers to the passage of the optic nerve through the sclerotic coat. This has usually been described as if the nervous fibres were transmitted through small perforations in a membrane, which has been called the *lamina cribrosa*. This notion of a particular membrane for transmitting the optic nerve in divided portions, is asserted by Dr. Jacob to have arisen from examining the part after the retina had been washed away, and the optic nerve cut off on the outside close to the sclerotic coat; the lamina cribrosa appearing to be nothing else than the extremity of the optic nerve. We are informed that, if the sheathing of the nerve be slit up through the sclerotic into the ball of the eye, the nerve does not present the appearance of a

---

* *Revue Medicale*, September.

† *Anatomical and Physiological Commentaries.* By Herbert Mayo, Surgeon and Lecturer on Anatomy. No. 11. July 1823.

‡ *Medico-Chirurgical Transactions*, vol. xii. part ii.

rounded apex, but that it is quite continuous, without any line of separation. If the optic nerve be pressed, the medullary matter is forced through the orifices at its extremity in "vermicular coils;" and a similar appearance may be produced upon the cut end of every part of the nerve external to the skull. Soemmering and other anatomists have represented a button, or tuft, projecting in the bottom of the eye. This likewise, according to Dr. Jacob, is artificially produced. He states that after death, especially if the parts be placed for some time in water, the medullary matter of the nerve protrudes, owing either to the neurilema contracting, or else to the medullary matter swelling from the absorption of water, so as to become too bulky for the structure by which it is surrounded. In reptiles and in fishes, where the nerve is not enclosed in a cylinder of unyielding texture, instead of a button or tuft, is found a "stellated or crucial" projection.

With regard to the termination of the retina anteriorly, very different opinions have been entertained. In the paper before us it is thus described: On removing the choroid, ciliary processes and iris, the retina is seen to terminate about a quarter of an inch from the circumference of the lens in a defined margin; the portion between this line and the lens, presenting the vitreous humour, retaining upon its surface some of the pigment which covered the ciliary processes. If the black pigment be removed from this part of the vitreous humour with a camel's-hair pencil, soon after death, there is produced an appearance as if the vascular layer was continued to the lens. But, after the parts have been allowed to remain in water twenty-four hours, the retina may be separated with very little force, and is even "frequently detached by the disturbance given in making the examination." If the choroid be removed without the retina, and the parts be kept in water for some days, the retina may be detached completely by simple agitation, the vascular layer remaining firmly attached at the line of termination above alluded to.

"The circumstance which has most strengthened the notion of the retina being continued forward to the lens, is that often, on raising the choroid and ciliary processes from the vitreous humour, we find those processes covered in several places by a fine semi-transparent membrane insinuated between the folds: this is supposed to be the vascular layer of the retina, but is really the corresponding part of the hyaloid membrane which is torn up, being firmly united to this part of the choroid. If the sclerotic, choroid, iris, and retina, be removed one or two days after death, leaving the vitreous humour with the lens imbedded on its anterior part, we observe a number of striæ on the vitreous humour converging toward the circumference of the lens, corresponding in number, size, and form, to the ciliary processes, giving the same appearance collectively that the circle of ciliary processes, or corpus ciliare, does on the choroid, and narrowed toward the nasal side, as the corpus ciliare is. This appearance has been noticed by most authors, but some describe it as arising merely from the marks left by the ciliary processes, while others consider these striæ of the same nature as those productions of the choroid, and call them the ciliary processes of the vitreous humour: it is the *corona ciliaris* of Camper and Zinn. If we remove the black pigment with a camel-hair pencil, we leave those productions on the vitreous humour more distinctly marked than when covered by the colouring matter, and presenting all the characters above stated; commencing behind with a well-defined margin, and terminating anteriorly by attachment to the capsule of the lens, the furrows between them capable of receiving the ciliary processes of the choroid, and the folds calculated to be lodged in the corresponding furrows of these processes. * * * If the cornea and iris be removed from a human eye within a few hours after death, a dark circle surrounding the lens, between it and the anterior extremities of the ciliary processes, may be observed: this is the part of the corona ciliaris of the vitreous humour, to which the ciliary processes of the choroid do not extend, which appears dark on account of its perfect transparency. The converging striæ are evident, even on this part where the ciliary processes are not insinuated, interrupting the view if we attempt to look into the bottom of the eye by the side of the lens. It is in my opinion, therefore, certain that part of the vitreous humour, as that transparent body is called, enters into the formation of the posterior chamber of the aqueous humour. * * *

"Each fold of the corona ciliaris of the vitreous humour seems to consist of two layers of hyaloid membrane, capable of being separated one from the other, and distended by inflation, and admitting of communication with each other round the lens. It appears to me that the canal of Petit, or *canal godronné*, is formed in consequence of these folds receiving the injected air one from the other: it is, however, generally described as being formed by the membrane of the vitreous humour splitting at the circumference of the lens, one layer going before and the other behind that body, the canal existing between these two layers and the capsule of the lens. That the capsule of the lens has no share in the formation of the canal of Petit, I conclude from filling this canal with air, and allowing the part to remain for some days in water, and then with great care removing the lens included in its capsule: this I do not find, however, causes the air to escape from these cells, but leaves them presenting nearly the original appearance; and, after the air has escaped, I can pass a small probe all round in this canal, raising by this means the folds from the hyaloid membrane. It is difficult, however, to preserve the air in these folds for any length of time under water, because the tendency of the air to ascend causes the rupture of the membrane, by which it is allowed to escape. After the lens, included in its proper capsule, has been detached from its situation on the vitreous humour, the space it occupied presents the appearance of a circular depression, surrounded by those productions of the hyaloid membrane of which I have just spoken; the vitreous humour remaining in every respect perfect, notwithstanding this abstraction of the lens."

The capsule of the chrystalline lens is regarded as cartilaginous,—first, from its elasticity, which enables it to assume a particular appearance when the lens has been removed, so that it does not fall into loose folds like other membranes; secondly, from the density of its texture; and, thirdly, from the permanence of its transparency. The lens has been considered by many writers as unconnected with its capsule. This opinion Dr. Jacob has endeavoured to disprove by the following method:—He removed the cornea and iris from an eye within a few hours after death, and placed it in water; he then divided the capsule all round, at the circumference of the lens, making the division behind the anterior convexity in such a manner that the lens could not be retained by any part of the capsule supporting it in front. He then inverted the eye, holding it by the optic nerve, when he found that the lens, if the eye was sufficiently fresh, could not be displaced by agitation. In the eye of a young man about six hours after death, he found that he could raise the eye from the bottom of the vessel by pushing a cataract-needle into the lens, after the anterior part of the capsule had been removed. The membrane which has been described as lining the inside of the cornea, and considered as belonging to the aqueous humour, is asserted to be cartilaginous, resembling in nature the capsule of the lens, and performing an analogous function,—viz. enabling the parts to preserve their proper shape.

Soemmering was the first to observe, and to describe, the appearance of a hole in the retina of the human eye, with a yellow margin, and a fold which occupies the situation of the hole, and tends to conceal it. Soemmering and others seem to regard the fold as the consequence of changes which take place after death. Dr. Jacob first inquires into the nature of such changes. If the eye has become flaccid before it is examined, the retina will be found to present an irregular surface, owing to a number of folds produced from the loss of the customary support, as is proved by the fact that these folds entirely disappear if the eye be immersed in water sufficiently long to allow the vitreous humour to become distended from its absorption. These accidental folds may readily be distinguished from the one in question. When the examination is made from without, by removing the posterior part of the sclerotic and choroid coats, the retina appears drawn, at one particular point, into the vitreous humour, the entire fold being rather more than the eighth of an inch in length. After the eye has continued for some time in water, the fold begins to yield, and a small slit is perceived, which gradually widens into a round hole, which is larger in proportion as the fold has yielded; and, when this has entirely given way, the appearance is presented of a hole with a yellow margin. If the examination be made by looking through the vitreous humour, the appearance of a hole is more remarkably conspicuous: that part of the retina is nevertheless seen to project. Dr. Jacob continues—

"In the eye of a young man, which I had an opportunity of examining under peculiarly favourable circumstances, within five hours after death, I noticed the following appearances. The cornea and iris having been cut away, and the lens removed from its situation, I placed the part in water beneath one of the globular glasses, and held it so as to allow the strong light of a mid-day sun to fall directly upon it, when the retina, to the outside of the optic nerve, presented unequivocally the appearance of being drawn or folded into the form of a cross or star, with a dark speck in the centre, surrounded by a pale yellow areola. I further satisfied myself of the prominence of the fold, by holding a needle opposite to it while the light shone full upon it; a shadow being thus cast upon the retina, which deviated from a straight line when passed over the situation of the fold. To ascertain whether there is actually a hole in the retina, or merely a deficiency of nervous matter at this point, I allowed the eye to remain for some days in water, until the connexions of the parts began to give way. I then introduced a small probe between the retina and vitreous humour, the part still remaining in water, and, bringing the blunt point of the instrument opposite the transparent spot, attempted to pass it through, but found I could not do so without using force sufficient to tear the membrane. I also removed the nervous matter by maceration and agitation in water; and, on floating the vascular layer, found that I could no longer ascertain where the spot had originally existed, there being no hole in the situation previously occupied by the transparent speck."

Some observations have been made by the same anatomist on the structure of the iris. If this be examined, either in the living body or under water in the dead subject, a number of irregular masses are seen projecting from the middle space, between the circumference and the pupil: from these, elevated lines, equally irregular, run towards the pupil, and are attached about the twentieth part of an inch from its margin. From this point many smaller striæ converge to the edge of the pupil. Of these we can attempt no description; it being declared by the author, that "it is quite impossible for words to give an adequate idea of this appearance:" we must therefore refer our readers to the plates which accompany Dr. Jacob's paper. We shall only remark, in addition, that he regards the iris as muscular, in common with most anatomists.

The membrana pupillaris has generally been supposed to disappear about the seventh month; but it is stated that this occurrence does not take place till about the period of birth.

In our Proëmium for July, we gave a short notice of some views of Mr. Charles Bell, with respect to the *physiology of the eye:* since that time his papers on the subject have been published in the Philosophical Transactions, and we are now enabled to give our readers a more detailed account of these very interesting investigations. Mr. Bell is of opinion that the attention of preceding inquirers has been too exclusively directed to the eye itself, without duly considering the external apparatus by which its various and complicated motions are effected; "yet this framework, or apparatus, is not less calculated to renew our wonder, than the properties of the organ itself." The paper is divided into two parts; the first of which treats of the uses of the apparatus exterior to the eyeball, and the second considers how the nerves minister to these functions.

The motions of the eye are shown to be two-fold,—viz. those for the direct purposes of vision, and those for the preservation of the organ. The first of these motions described by Mr. Bell, is one, which, he informs us, from its rapidity, has escaped observation; it is this:—at the instant the eyelids are brought together, the eyeball moves in such a manner as to throw the cornea upwards under the projection of the upper eyelid; and this takes place even during the most rapid winking.

The movement here described may be experimentally proved by fixing one eye upon an object, and closing the other with the finger, so as to feel its convexity through the eyelid; the other eye is then to be closed, when it will be perceived that the cornea of the eye first shut is immediately elevated. "When a dog was deprived of the power of closing the eyelids of one eye by the division of the nerve of the eyelids, the eye did not cease to turn up when he was threatened, and when he winked with the eyelids of the other side." A circumstance nearly similar was noticed by Mr. Bell in a girl who had lost the power of closing the eyelids in consequence of a burn: when she would have winked, the cornea was turned upwards, so as to come into contact with the mouths of the lacrymal ducts, and was thus kept moist. When the eyelids meet, their margins touch only at the outer edge, by which means a small gutter is left between them and the surface of the cornea. Now, if the eyeball remained stationary, that portion of it which is directly in the very axis of vision would be left untouched, and would even be suffused with tears: hence the purpose of this revolving movement, which sweeps the surface of the cornea. Another purpose served by this arrangement is, that it facilitates the flow of tears from the lacrymal ducts; the simultaneous ascent of the cornea and descent of the eyelid stretching the membrane on which the mouths of the ducts open, and producing an effect similar to the elongation of the nipple. With regard to the eyelids themselves, it is to be remarked that, while the upper one rises and falls perpendicularly, the lower moves towards the nose, playing horizontally to a certain extent. This is part of a provision for propelling particles of dust, &c. towards the inner canthus.

We formerly mentioned (Proëmium for July,) the remarks of Mr. Bell on the state of the eye during sleep, fainting, and the approach of death; and his division of the muscles into voluntary and involuntary, the recti belonging to the former, and the oblique to the latter class. The following are the experiments on which the reasonings of Mr. Bell are founded:*

"1. I divided the superior rectus or *attollens* in a rabbit, and felt something like disappointment on observing the eye remain stationary. Shortly afterwards, on looking to the animal while it was feeding, I saw the pupil depressed, and that the animal had no power of raising it.

"The explanation I conceive to be this: During the experiment the eye was spasmodically fixed by the general action of the muscles, and particularly by the powerful retractor, a muscle peculiar to quadrupeds. But, on the spasm relaxing, and when the eye was restored to the influence of the voluntary muscles, the recti, the voluntary power of raising the eye being lost by the division of the superior muscle, the eye was permanently depressed.

"2. Wishing to ascertain if the oblique muscles contract to force the eyeball laterally towards the nose, I put a fine thread round the tendon of the superior oblique muscle of a rabbit, and appended a glass bead to it of a weight to draw out the tendon a little. On touching the eye with a feather, I had the pleasure of seeing the bead drawn up; and,

* *On the Motions of the Eye, in illustration of the Uses of Muscles and Nerves of the Orbit;* by C. Bell, Esq.—(*Philosophical Transactions,* 1823.)

on repeating the experiment, the thread was forcibly drawn through my fingers.

"By experiments made carefully in the dead body, (having distended the eyeball by dropping mercury into it to give its full globular figure,) I had found that the action of the superior oblique muscle is to turn the pupil downwards and outwards, and that the inferior oblique just reverses this motion of the eye. In the above experiment there is abundance of proof that the superior oblique muscle acted, and yet the pupil was not turned downwards and outwards; therefore both oblique muscles must have been in action. Their combined action draws the eyeball towards the nose.

"In the violent spasmodic affection of the eye, when it is painfully irritated, I believe that all the muscles, both of the eyeball and eyelids, are excited. In quadrupeds, I have ascertained that the oblique muscles act when the haw is protruded; but I have also found that the retractor oculi, alone, is capable of forcing forwards the haw.

"But quadrupeds, having an additional apparatus of muscles to those of the human eye, are not suited for experiments intended to illustrate the motions of our eyes. The monkey has the same muscles of the eye with man.

"3. I cut across the tendon of the superior oblique muscle of the right eye of a monkey. He was very little disturbed by this experiment, and turned round his eyes with his characteristic inquiring looks, as if nothing had happened to affect the eye.

"4. I divided the lower oblique muscle of the eye of a monkey. The eye was not, in any sensible manner, affected; the voluntary motions were perfect after the operation.

"5. On holding open the eyes of the monkey which had the superior oblique muscle of the right eye divided, and waving the hand before him, the right eye turned upwards and inwards, while the other eye had a scarcely perceptible motion in the same direction. When the right eye was thus turned up, he seemed to have a difficulty in bringing it down again.

"From these experiments it is proved, that the division of the oblique muscles does not in any degree affect the voluntary motions by which the eye is directed to objects.

"This cannot, however, be said of the involuntary winking motions of the eyes. We have seen that, in winking to avoid injury, the oblique muscles were in operation; and that the inferior oblique muscle gained in the power of elevating the eyeball by the division of the superior oblique, its opponent."*

* "Since this paper was read, a case has occurred in the Middlesex Hospital, under the care of my colleague, Dr. Macmichael, which shows the consequences of the eye and eyelids being rendered immoveable. In this case the surface of the eye is totally insensible, and the eye remains fixed, and directed straight forwards, whilst the vision is entire. The outward apparatus being without sensibility and motion, and the surface not cleared of irritating particles, inflammation has taken place, and the cornea is becoming opaque; thus proving the necessity of the motions of the eye to the preservation of the organ. Another curious circumstance, illustrative of the observations made above, is, that when both eyes are shut, the eye affected continues to be sensible of a red light coming through

During repose the voluntary muscles resign their office, and the involuntary muscles draw the pupil under the upper eyelid; but, when the eye is in action, we receive two distinct impressions: first, that upon the retina; and, secondly, that which conveys the idea of position, —this last depending solely upon the voluntary muscles. It is thus illustrated:—

"Let the eyes be fixed upon an illuminated object until the retina be fatigued, and in some measure exhausted by the image, then closing the eyes, the figure of the object will continue present to them: and it is quite clear that nothing can change the place of this impression on the retina. But, notwithstanding that the impression on the retina cannot be changed, the idea thence arising may. For, by an exertion of the voluntary muscles of the eyeball, the body seen will appear to change its place, and it will, to our feeling, assume different positions according to the muscle which is exercised. If we raise the pupil, we shall see the body elevated, or if we depress the pupil, we shall see the body placed below us; and all this takes place while the eyelids are shut, and when no new impression is conveyed to the retina. The state of the retina is here associated with a consciousness of muscular exertion; and it shows that vision, in its extended sense, is a compound operation, the idea of position of an object having relation to the activity of the muscles.

"We may also show, by varying the experiment, that an agitated state of the muscles, or a state of action where the muscles are at variance or confused, affects the idea of the image. If we look on the luminous body so as to make this impression on the retina, and then cover the face so as to exclude the light, keeping the eyelids open, and if we now squint or distort the eyes, the image, which was vividly impressed upon the retina, instantly disappears, as if it were wiped out. Does not this circumstance take place because the condition of the muscles, thus unnaturally produced, being incongruous with the exercise of the retina, disturbs its operation?

"If we move the eye by the voluntary muscles, while this impression continues on the retina, we shall have the notion of place or relation raised in the mind; but, if the motion of the eyeball be produced by any other cause, by the involuntary muscles, or by pressure from without, we shall have no corresponding change of sensation.

"If we make the impression on the retina in the manner described, and shut the eyes, the image will not be elevated, although the pupils be actually raised, as it is their condition to be when the eyes are shut, because there is here no sense of voluntary exertion. If we sit at some distance from a lamp which has a cover of ground glass, and fix the eye on the centre of it, and then shut the eye and contemplate the phantom in the eye; and if, while the image continues to be present of a fine blue colour, we press the eye aside with the finger, we shall not move

---

the eyelid, whilst the sound eye is in darkness. The reason of this I apprehend to be: the eye which possesses its natural motions is turned up, but the eye which continues fixed, looking forwards, receives the light through the transparent eyelid; and thus it appears that the dropping of the eyelid would make an imperfect curtain, if unaccompanied by the turning-up of the eyeball during repose."

that phantom or image, although the circle of light produced by the pressure of the finger against the eyeball moves with the motion of the finger.

"May not this be accounted for in this manner:—The motion produced in the eyeball not being performed by the appropriate organs, the voluntary muscles, it conveys no sensation of change to the sensorium, and is not associated with the impression on the retina, so as to affect the idea excited in the mind? It is owing to the same cause that, when looking on the lamp, by pressing one eye we can make two images, and we can make the one move over the other. But, if we have received the impression on the retina, so as to leave the phantom visible when the eyelids are shut, we cannot, by pressing one eye, produce any such effect. We cannot, by any degree of pressure, make that image appear to move, but, the instant that the eye moves by its voluntary muscles, the image changes its place: that is, we produce the two sensations necessary to raise this idea in the mind; we have the sensation on the retina combined with the consciousness or sensation of muscular activity."

In considering the nerves of the eye, it is proper to keep in mind that the sensibilities of different parts of the body differ as much in kind as in degree. The fifth pair gives to the surfaces of the head and face the same kind of sensibility which the spinal nerves communicate to the rest of the body; but, besides this, some of its branches also bestow "that distinct sense on certain parts for the purpose of drawing the muscles into combination;" an example of which is the acute sensibility of the surface of the eyeball to minute particles of foreign bodies, which both excites the flow of tears, and draws the muscles into combined action to expel the offending matters. On dividing the branch of the fifth which goes to the cheek and lips, these become deprived of their sensibility, although expressly supplied with other nerves, and retaining the power of motion; so, likewise, if that branch which goes to the forehead be divided, the sensibility of the parts is lost: hence we are led to the conclusion, "that all the branches of the same division resemble each other in function, and bestow sensibility on the parts within as well as on those without." That the ophthalmic nerve may have its function destroyed, and the sensibility of the parts supplied by it lost in consequence, we learn from the following case, communicated to the author by Mr. Crampton, of Dublin. In perusing this case, it is necessary to keep in mind that this nerve supplies the parts within the orbit, and thence ramifies upon the eyelids and forehead.

"A few days after the discharge from the ear had ceased, the eye became entirely insensible to the touch. This loss of feeling extended to the lining of the eyelids, to the skin covering them, and to the skin of the cheek and forehead, for about an inch surrounding the eye: it did not go beyond the middle line of the face. When she told me her eye was *dead*, (as she expressed it,) to be certain, I drew my finger over its surface; and, so far was this from giving her pain, that she assured me she could not feel that I was touching it at all. The eyelids made no effort to close while I was doing this, but the conjunctiva appeared sensible to the stimulus, as a number of vessels on the surface of the eye became immediately injected with blood."

"Here (continues Mr. Bell,) we have an insensibility of the eye itself corresponding with the insensibility of the skin, which latter part we know possesses sensibility through the fifth nerve; and we therefore conclude that it is the affection of the same nerve near its root to which we have to attribute the insensibility of the surfaces of the eye, as well as of the skin around the eye.

"By experiment it can farther be made evident, that the sensibility of the eye, enjoyed through the ophthalmic nerve, does not bestow on the organ directly the power of combining the muscles, either for the defence of the eye or for any other purpose. The impression must be referred back to the brain, and the muscles excited by their proper nerves. I have not been able to excite the motion of the eye by irritating the ophthalmic division of the fifth after the division of its root,* and in the instance just given the eyelids did not move when the surface of the eye was irritated, because no sensation was conveyed inward to the sensorium, and consequently no mandate transmitted from it. The young lady could see, and could move the eye and eyelids; the eye itself was irritated by touch, as appeared from the rising inflammation; but, by the insensibility of the ophthalmic nerve, a link was lost in the relation necessary to join the action of the muscles to the sensibility of the surface."

The motions of the eyelids, both voluntary and involuntary, are described as depending upon the *respiratory nerve* of the face: it contracts the eyelids at will, and also produces the effect of involuntary winking. The rolling of the eyeball, which takes place sympathetically with the closing of the lids, is effected through the influence of the fourth nerve. This rises at a part of the brain remote from the other nerves of the orbit, runs along without touching any of them, and is entirely distributed upon the superior oblique; a peculiarity which exists in all animals. The nerve in question takes its rise from the same tract of fibrous substance which gives origin to the respiratory of the face: "in a word, the connexion of the root declares the office of this nerve,"—viz. to effect the rolling movement of the eyeball, and to associate this with the winking of the eyelids. The third and sixth are the voluntary nerves, both being distributed to the muscles within the orbit, and conveying the perceptions of place or rotation. Mr. Bell concludes this part of the subject with the following remarks:

"I hope I have now unravelled the intricacy of the nerves of the head, and have correctly assigned to each nerve its proper office. In our books of Anatomy, the nerves are numbered according to the method of Willis, an arrangement which was made in ignorance of the distinct functions of the nerves, and merely in correspondence with the order of succession in which they appear on dissection.

"The first nerve is provided with a sensibility to effluvia, and is properly called olfactory nerve.

---

* "In attempting to excite the muscles of the eye by galvanism sent through the fifth nerve, the muscles of the jaw were affected."

"The second is the optic nerve, and all impressions upon it excite only sensations of light.

"The third nerve goes to the muscles of the eye solely, and is a voluntary nerve by which the eye is directed to objects.

"The fourth nerve performs the insensible traversing motions of the eyeball. It combines the motions of the eyeball and eyelids, and connects the eye with the respiratory system.

"The fifth is the universal nerve of sensation to the head and face, to the skin, to the surfaces of the eye, the cavities of the nose, the mouth, and tongue.

"The sixth nerve is a muscular and voluntary nerve of the eye.

"The seventh is the auditory nerve; and the division of it, called *portio dura*, is the motor nerve of the face and eyelids, and the respiratory nerve, and that on which the expression of the face depends.

"The eighth, and the accessory nerve, are respiratory nerves.

"The ninth nerve is the motor of the tongue.

"The tenth is the first of the spinal nerves: it has a double root and a double office; it is both a muscular and a sensitive nerve.

"Had I taken the nerves of any other complex organ rather than of the eye, I should have had an easier task. If I had taken the nerves of the tongue, I should have been able to prove by experiment, and in a manner the most direct, that the three nerves belong to three distinct functions, and stand related to three different classes of parts. I could have shown that taste and sensibility belong to the office of the fifth nerve, voluntary motion to the ninth, and deglutition to the glosso-pharyngeal nerve of the tongue."

The *comparative anatomy and physiology of the organs of vision* have been investigated with much attention by M. TREVIRANUS,* who has brought together some facts, either altogether new, or else too much overlooked. The eyes of insects are either simple or compound, and the author had shown, in his former memoirs, that the compound eyes consist in reality of an assemblage of simple eyes situated close to each other; the only difference between these last and the facets of the first is, that each simple eye receives a proper nerve from a particular point of the brain, while the compound eye receives a bundle consisting of as many fibres as there are corneas. Now insects present us here with a remarkable difference: in some this mass is composed of nervous fibres, which pass through a membranous partition, covered with a coloured pigment, and which may be compared to the choroid, and each of which terminates on the posterior surface of each division of the compound eye, after its sheath and anterior extremity have received a coating of this coloured pigment. The cornea is likewise covered, on all its internal face, with a matter, the colour of which frequently differs from that of the pigment. CUVIER, in dissecting the eyes of insects, only found this kind, which he has therefore attributed to the

---

* *Observations pour servir de Complement à l'Anatomie, comparée et à la Physiologie des Organes de la Vue.* Par le Docteur GODEFROI-REINHOLD TREVIRANUS, Professeur à Brême. (*Journal Complémentaire,* October.)

entire class.* M. MARCEL DE SERRES remarked, that the choroid and the pigment are wanting in certain insects, while in others the pigment of the cornea is penetrated by the fibres of the optic nerves, and that their extremities are exposed naked to the light.† The observations of M. Treviranus do not confirm this last assertion; and the structure of the simple eyes of insects likewise affords an analogy against it. De Serres alleges, in proof of his opinion, that, after having removed the cornea with care, the fibres of the nerve may be seen projecting through the pigment in the form of raised white points; but, according to Treviranus, when this proceeding is adopted, the colouring matter which lines the internal face of the cornea, as well as the extremities of the optic nerve, remains adherent to the cornea; and, consequently, the extreme nervous fibres present this appearance from having had the pigment violently removed. The latter author maintains that, after removing the cornea, the extremity of the subjacent nerve is always found covered with pigment, except where there has been an evident laceration. In the *blatta orientalis,* however, and others of the same class, some peculiarity of structure is observable. In this insect, the transparent substance is found between the extremity of each fibre of the optic nerve, and the corresponding section of the cornea. Beneath the cornea of the compound eye, a mass of a deep violet colour is found, which, examined with the microscope, appears to be an aggregate of as many pyramidal bodies as there are divisions in the eye: each division has its separate pyramid, the rounded base of which adheres to it. These pyramidal bodies are composed of two substances,—viz. of a mass analogous to the vitreous humour, and a violet pigment which covers its sides: this pigment is wanting at the base where it rests immediately upon the cornea, and the optic nerves are distributed to the extremities of these bodies, in the form of fibres. The insects having the cornea lined with a coloured pigment, have nothing resembling eyelid or iris,—nothing which hinders the light from penetrating into the eye, and therefore require this contrivance to protect the nerves against the direct application of the light. But the others, the structure of whose eyes has just been described, require an organization by which the impression of the light may rather be increased than diminished: hence the vitreous body between each division of the cornea, and the corresponding filament of the optic nerve. Some insects which range only in the light, as the day butterflies, have a pigment so thick beneath the cornea, that it is difficult to imagine how the rays can penetrate it. M. Treviranus, however, is of opinion that the opacity is generally increased after death, particularly if the insect has been immersed in spirit of wine, as is frequently the case before the examination is made. Indeed, we must either suppose it sufficiently transparent during life to admit the light, or else agree with M. de Serres that the filaments of the nerve present against the inner surface of the cornea without any covering.

The passage of the light through a coloured pigment must necessarily

---

* *Leçons d'Anatomie comparée,* tom. ii.

† *Memoires sur les Yeux composés, et les Yeux lisses des Insectes.*

deprive the animal of the power of distinguishing colours, all external objects appearing in this respect like that medium through which the rays pass. The colour of the pigment varies in different species. A more important fact is, that in certain insects no image of the visible object is formed; the representation of external bodies at the bottom of the eyes is not, therefore, a necessary part of vision in general, but only of particular kinds of vision.* In the one case, the eye may be compared to a camera obscura, and in the other to a convex mirror, in which the objects are represented in a magnified image. The surface of the entire cornea receives the images of large and distant objects, while the divisions receive those which are minute or near: the former are perceived by the optic nerve at large, and the latter by individual ramifications.

A considerable analogy seems to exist in some classes between the senses of sight and touch; the only essential difference between the nerves of the two senses being, that those of touch are expanded beneath an opaque, and those of sight beneath a transparent, pellicle In some the analogy is very striking. The snail *(helix pomacia)* carries at the summit of its large horns an eye, composed of a cornea and a transparent gelatinous mass, situated behind the horn, and on which the optic nerve is distributed. In the black slug *(limax acer)* an optic nerve, exactly similar, is found, and with a similar distribution, except that it does not ramify behind a transparent organ, but behind a black opaque membrane: notwithstanding this circumstance, however, the slug receives impressions from distant objects by means of its horns, as well as the snail, without requiring to touch them.

Some interesting observations follow upon the visual organs of fishes, which our limits forbid us to enter upon. The iris in some birds, particularly the *falco tinnunculus,* was subjected to minute microscopic examination; the results of which seem to confirm the opinions of M. Maunoir with regard to the structure of this part. Maunoir thought he could perceive, by the aid of a good glass, muscular fibres forming two concentric circles,—one placed on the inner edge of the membrane round the pupil, and composed of circular fibres; the other external, and having the fibres directed from the great circumference towards the small. M. Treviranus found a glass which magnified but little, sufficient to detect some appearance of fibres in the iris; one, which magnified 150 times, discovered true bundles of fibres; one, which magnified 300 times, showed the same transverse and annular striæ which are perceptible in the fibres of muscles. He infers, therefore, that the iris in birds is muscular, but that the direction of the fibres differs considerably from the description of M. Maunoir. The observations of the latter, we must remember, were made on mammiferous animals. In the *falco tinnunculus*, the fibres are parallel to the great, and not to the small, circumference; no radiating ones were discovered; and the iris itself was far from being composed entirely of fibres of any kind, the larger portion of it consisting of a spongy cellular texture.

---

* See a paper in the *Annals of Philosophy*, for July 1817.

Scarpa has described the axis of the cochlea of the *ear*, when it is cut longitudinally, as composed of two substances,—one tubular, and very friable, the other hard and compact. M. Rosenthal says, that the axis of the cochlea is indeed formed of an internal solid substance, and an external one which is friable; but, he adds, that this exterior tubular pellicle belongs more to the spiral lamina than to the axis, and that it is separated from the more compact mass by a considerable canal. This tubular pellicle ascends into each turn of the internal scala, separated by an interval from the more compact substance of the axis, and gains the spiral lamina, on the inferior border of which it is lost. This interval forms a canal, which turns round the axis even to the infundibulum, so that this canal follows exactly the tractus spiralis foraminulentus, which is found in the bottom of the common nervous canal. It results from this, that all the nervous filaments which pass by the holes of this tractus or furrow introduce themselves there, and that they are spread in fine threads over the spiral lamina. The filaments destined for the first turn, mount upwards, applied immediately to the interior tubular lamina; those of the second turn are conducted by this canal to the tubular substance of the spiral lamina which belongs to them. The situation of this canal, and its great size, sufficiently indicate, according to M. Rosenthal, that other writers have been wrong in taking it for the separation of the plates of the spiral lamina. He adds, that it is not nearly of the same size in all persons, and that it is not improbable that this difference may influence the nervous energy; for the result of numerous dissections has convinced him that very slight shades of difference in the structure of this organ were sufficient to derange its functions.*

An interesting peculiarity of structure has been observed in singing birds, by Mr. Clayton and Dr. Moulin.† It appears from their account, (and which we take from the Journal of Foreign Science,) that a passage exists by which the ears communicate, so that water passes from the one to the other when the tympanum on both sides is pierced. Two plates of bone constitute, as it were, a double skull, being placed so as to leave a cavity between them. The outer table is supported by numerous capillary columns. This passage of communication is so much larger in birds that sing, that they can be distinguished by a simple inspection of the head.

Notwithstanding the labour that has been bestowed by various anatomists on *the structure of the uterus*, the history of its nerves remains imperfect, and it is only very recently that they have been clearly demonstrated by the indefatigable Tiedeman, of Heidelberg, who has published a description of them, with plates in illustration.‡ The nerves of the uterus, ovaries, and fallopian tubes, according to this anatomist, come from the great sympathetic of either side, and form six plexuses. The first he calls the *spermatic*, or common plexus of the

* *Journal Complémentaire*, Aout.

† Clayton's *Letters from Virginia*.

‡ *Tabulæ Nervorum Uteri.* F. Tiedeman, M.D. &c. Heidelberg, 1822.

ovaries and fallopian tubes: it is situated on the anterior face of the abdominal aorta, at the root of the internal spermatic artery. Composed of many filaments which come from the renal nerves of both sides, it descends into the pelvis with the spermatic artery, and goes to distribute itself principally on the ovary and fallopian tube, although a few branches are likewise sent to the uterus. The second, which is the most important of the whole, is denominated the *great superior lumbar plexus,* or common uterine: it arises from the renal and superior lumbar ganglions of the great sympathetic; it is situated before the fifth lumbar vertebra, before the common iliac arteries, and extends to the promontory; there it divides into two portions, which penetrate into the pelvis, where they immediately form two plexuses, which are named by M. Tiedeman the *hypogastric or lateral uterine,* and to which some threads run from the first and second sacral ganglions. Each of these plexuses sends many filaments to the uterus; they accompany the uterine artery, with the ramifications of which they penetrate the substance of that organ. From the sides of the superior lateral hypogastric plexus, many branches go off to the neck of the uterus and vagina, and which, having united with the anterior branches of the third and fourth sacral nerves, form a grand plexus, which the author calls the *inferior lateral hypogastric.* This plexus supplies branches to the uterus, vagina, bladder, and rectum; the ramifications always accompany the uterine artery, which they surround like net-work.

From this account it appears, according to Tiedeman, that the great sympathetic sends many nerves to the uterus, instead of this organ receiving only very small branches, or being destitute of any; opinions, both of which had been advanced. The author adds, that the nerves are small, soft, and reddish, being applied to the coats of the arteries; on entering the substance of the uterus, they disappear all at once, and cannot be distinguished, even with the assistance of a good glass: they seem to terminate in the cellular or mucous texture intermediate between the blood-vessels and lymphatics, no less than between the fleshy fibres, or even to be converted into these. Accordingly, M. Tiedeman is among those anatomists who think that the ramifications of the great sympathetic nerve ought to be considered as belonging exclusively to the arteries.

However interesting these observations may appear, the same distinguished anatomist has established other points of no less importance: thus, he states that the number and size of the uterine nerves vary at different periods of life; that they are very small in young girls, larger at the age of puberty, and again become very small in old women. He has likewise found, by the examination of many women who had died a short time after their delivery, that these nerves really become larger and more numerous during the period of utero-gestation,—as stated by Hunter. The plates which are intended to illustrate the description are said to be extremely well executed.*

A very full and interesting history of the *great sympathetic nerve*

---

* *Journal Complémentaire,* 91 *cahier.*

has been published by M. LOBSTEIN.* He adopts the general view of its being a peculiar nervous centre, independent of the cerebrum and spinal cord, and enters minutely into the anatomy, physiology, and pathology of this system. We have thought it advisable to give an analysis of this important work, and therefore shall not attempt any account of it in this place.

MM. PREVOST and DUMAS have engaged in some *researches into the nature of the blood,* and its action in the different phenomena of life, which seem likely to be attended with interesting results.† They regard this fluid as nothing else than serum, holding in suspension small, regular, insoluble particles. These are stated to appear, when examined through the microscope, to be uniformly composed of a central spheroid, which is colourless, and a membranous bag, which is red and surrounds the spheroid; from which, however, it is easily separated after death. The central substance is white, transparent, and of a spherical form, in animals which have circular particles, and oval in those which have elliptical particles; the diameter is uniform in the first, but varies perceptibly in the second. The coloured part appears to be a kind of jelly, which is easily denuded, but which is insoluble in water, from which it may always be separated by repose. These bodies have a great tendency to form aggregates, like strings of beads; and as the force which keeps the red substance fixed round the globules ceases at the same time with the movement of the liquid, they then form a net-work, in the meshes of which the liberated red colouring matter becomes enclosed, as well as many particles which do not undergo this separation. This mass, called in common language the clot or cruor, gradually allows the liquid it had imprisoned to escape, and sinks to the bottom from its weight. No other change takes place, unless some alteration be made in its texture; but, if it be torn and exposed to the action of water, this takes away the liberated colouring matter and the particles which have remained entire, while the white globules remain on the cloth, or searce used, in the form of filaments, and constitute the *fibrine.* This distribution of the materials of the blood perfectly explains the failure of all attempts made to insolate the colouring matter. The three substances which are most worthy of attention in the chemical history of the blood, are the albumen of the serum, the white globule, and the colouring matter in which this is enveloped. Albumen, when coagulated, exhibits the same white globules which have been described above: they consider its coagulation by alcohol as the best method of obtaining it in a state of purity; and, when in this manner it is subjected to examination and chemical tests, it is found to differ in nothing from fibrine. The colouring matter has engaged the attention of many eminent chemists, who all appear to have been misled by a very simple circumstance. It is extremely divisible, without being soluble in water;

* *De Nervi Sympathetici Humani, fabrica, usu, et morbis; Commentatio Anatomico-Physiologico-Pathologica, &c. &c.* Auctore J. F. LOBSTEIN, &c.—Parisiis, 1823.

† *Annales de Chimie et de Physique*, xxiii.

it even passes through filters, but the fragments may be detected by means of the microscope, and they fall down in the form of a red deposit. The transparency of the water being undisturbed while it assumed a red colour, induced chemists to regard this substance as soluble, and hence they subjected the coloured liquor thus obtained to chemical tests, with results which have always proved unsatisfactory. The processes pointed out by Berzelius, Brande, and Vauquelin, for separating the colouring matter, are therefore illusory, according to the views of MM. Prevost and Dumas. This colouring matter appears to be formed of peroxide of iron, with some animal substance: this has been generally regarded as albumen, but the question is by no means set at rest. The clot is composed of all the corpuscles, and a quantity of serum more or less considerable according to the period during which it has been left undisturbed, and contains no other matters of any kind, except in a state of disease. The serum, on the other hand, varies in the same animal, and also in different animals, in such a manner as to render it impossible to connect any physiological characters with its condition. The following general conclusions are deduced:—1. That arterial blood contains more corpuscles than venous blood; 2, that birds have the greatest number of these corpuscles in their blood; 3, that the mammifera come next in order, and the carnivora appear to have more than the herbevora; 4, that cold-blooded animals possess the smallest number.

The next part of the observations of MM. Prevost and Dumas relates to the presence of certain principles in the blood, dependent upon diseased conditions of the fluid. This part of the subject is connected with the physiology of secretion. At first sight, it would appear that we could not appreciate the changes which take place in a secreting organ, without subjecting to analysis the blood going to the organ in question, the blood coming from it, and the product of the secerning process: a moment's reflection will show the impossibility of our ever obtaining these data, but there is nevertheless a satisfactory method of overcoming this difficulty in certain cases. The blood which is circulated through a secreting organ experiences certain changes in its passage, and is again received into the general mass: now, if by any means the secreting apparatus could be deprived of its influence, of course the blood would cease to undergo the specific changes. Each portion of this fluid would thus induce a change in the general circulating mass, which, although at first inappreciable, would nevertheless come to be perceptible by its accumulation; and, at the end of a certain time, it may be presumed that the whole blood in the circulation would come to resemble, in some degree, the portion which in the natural state flows to the secreting organ. The blood thus altered might then be subjected to analysis, and the result compared with the fluid in its healthy condition. All attempts, however, to paralyse the action of a secreting organ must necessarily be liable to objections, and the only unexceptionable plan is to extirpate the part in question, by which the conditions supposed above are fulfilled. Richerand had already examined the effects produced by ligatures applied to the ureters, and he found the secretion of urine continue: these tubes became gorged, and the subjects of experiment died within a few days of what he has called *urinary fever*. He then removed the kidneys, an experiment which was followed by interesting results: if only one was extirpated, the animal was not affected; but, when both were removed, the animals invariably died in the course of a few days. In these cases, the gall-bladder was found to be gorged; and this secretion appeared to M. Richerand to act as a substitute for that of the kidneys. MM. Prevost and Dumas repeated these experiments on various animals, particularly dogs and cats; rabbits being found to bear them very ill. The operation is represented as being possessed of no real difficulty: a lean animal is chosen, and an incision made through the parietes of the abdomen, which should commence at the inner third of the last rib, and be carried to an extent varying according to the size of the individual, along the inner edge of the quadratus lumbarum. The fore-finger of the left hand is to be introduced into the wound, taking care not to injure the peritoneum; the kidney is then gradually detached from its connexions, and extracted by means of a hook or polypus forceps, and is to be removed, a ligature being previously applied to its vessels. The muscles are to be kept in contact by a few stitches of suture, and the skin similarly treated; by this means hernia is prevented. We are directed, when we are desirous of performing these experiments, to extirpate the right kidney in the first instance, on account of its connexions with the liver, and then to allow an interval of fifteen days to intervene before we remove the other. The health of the animal is nowise affected by the first operation, if it be successfully performed, and the wound is generally cicatrised at the end of three days. When the other kidney is removed, the health is seldom affected before the third day, during which interval the wound has closed, and the animal regained its activity and liveliness; it eats and sleeps as usual, and drinks but little; the temperature, respiration, and pulse, do not vary in any decided manner. At the end of this time, copious liquid brown stools, and vomiting of similar matters, announce the disturbance of the system. The pulse becomes small and rapid, amounting sometimes to 200 in the minute; and the respiration becomes frequent, short, and, towards the conclusion, oppressed. Lastly, all these symptoms become aggravated, and the animal dies between the fifth and ninth day. On dissection, the following appearances are stated as constantly presenting themselves:—Effusions of clear limpid serum into the ventricles of the brain,—the quantity in a dog of the middle size occasionally amounting to an ounce; the bronchia contain a large quantity of mucus, and the lungs appear somewhat denser than natural; the liver appears inflamed to a greater or less extent; and the gall-bladder is filled with bile, of a greenish or deep-brown colour; the intestines contain a quantity of liquid fæces, of the same colour as the bile; the urinary bladder is strongly contracted.

MM. Prevost and Dumas, estimating the quantity of urea formed by a middle-sized dog at a drachm or more in the day, were induced to hope that the questions relative to the particular functions of the kidney might be decided by examining the blood of those in whom this organ had been extirpated. When the dogs which had been operated upon became so languishing as to render it probable they had not long to live, they were bled, and the blood subjected to careful analysis. In the first place, it was observed to be more serous than the blood of the same animals when in health, and the serum itself contained a larger proportion of water: this was to be expected, considering that cutaneous transpiration cannot, in these animals, supply the place of the action of the kidneys. The serum and clot were treated with boiling water, as long as this had any perceptible action upon them. The washings were evaporated, and subjected to alcohol, which dissolved the *muco-extractive* matter, described by Dr. Marcet. Healthy blood, similarly treated, yielded only half the quantity of this alcoholic residuum. In either case it was of a brown colour, soluble both in water and in alcohol, absorbing moisture from the atmosphere, and precipitating the acetate and nitrate of lead. That procured from animals deprived of their kidneys concreted with nitric acid into a white crystalline mass. These, and other characters, indicated the presence of a peculiar animal matter, capable of combining with oxide of lead, of urea, and of lactate of soda. The urea was next purified by converting the residuum into a nitrate, and leaving this compound upon unsized paper for some hours; by this means the whole of the lactate of soda was separated, deliquescing and sinking into the paper. On redissolving the nitrate in water, there remained a small residuum, which seemed to be a combination of nitric acid with the animal matter above alluded to. The evaporation of the liquid gave the nitrate of urea in white pearly scales. This was ascertained to be the same kind of urea as that obtained from urine.

The phenomena which are attendant on *muscular contraction* have likewise been ably illustrated by the same ingenious physiologists.* The muscles in the state of repose present the appearance of straight fasciculi of fibres, which are parallel, and united by cellular texture. If a muscle, sufficiently fine to retain its transparency, be placed under a microscope, and contractions be excited in it by means of a galvanic current, the fibres are seen to be instantly thrown into a zig-zag form, and this action produces the contraction of the muscle. This change in the shape, however, produces none in the bulk of the organ, as had already been shown by the experiments of Barzotelli. The nervous branches are distributed in the muscle, at first without observing any regular course; but, if these branches be followed, and examined under a sufficient magnifier, they will be found to spread themselves out, and to divide into separate filaments, which run parallel to one another, but in a direction at right angles to the muscular fibres. They are folded upon themselves after having run a little way, and return towards the point from which they set out, losing their parallelism by degrees, and re-entering the fasciculus from whence they originated. Frequently, instead of returning to the same fasciculus, they anastomose with a neighbouring one; but in every case the elementary nervous fibres tra-

---

* *Memoire sur les Phenomenes qui accompagnent la Contraction Musculaire.* Par MM. Prevost et Dumas.

verse the muscle, cutting its fibres at right angles. On these grounds, the phenomena attending muscular contraction are supposed explicable, by a galvanic current traversing the nervous filaments which are known to be good conductors, and to be well insolated by their fatty envelope. According to the law of M. Ampère, they will approach each other, carrying with them the muscular fibres to which they are attached, and will thus cause the plaiting or folding which has been described; and the shortening of the muscle must follow, as a necessary consequence. If this hypothesis be well-founded, the muscle will become a very sensible galvanometre. If the galvanic current be passed into an insolated portion of nerve, the muscle to which it goes will contract immediately, even although it may not be included in the circle. According to the hypothesis of MM. Prevost and Dumas, this result could not be explained if the nerve be regarded as a simple conductor; but it is easily understood if it be admitted that two opposite conductors exist in each nerve, as the result and anatomical structure would likewise seem to show.*

An interesting paper, on *the Process of Re-production in the Members of the Aquatic Salamander*, has been published by Dr. J. T. Todd.† When the limbs of this animal are removed, the appearances of inflammation present themselves, as in other vertebral animals; nor can any difference be perceived until the cicatrix is completely formed, except that the extremity of the stump becomes enlarged and bulbous. This swelling generally precedes the formation of the cicatrix, occasionally it is simultaneous, and very seldom occurs afterwards. A short time after the cicatrix has been formed, (from one day to six,) a red point is observed projecting at the centre, from which the cuticle has been removed: this point is soft, and its surface secretes a glutinous fluid, which adheres to the fingers on being touched; it is surrounded by a groove, formed by the cicatrix being elevated above the level of the bone. Microscopic observation proves this spot to be a cluster of blood-vessels, which has protruded through the cicatrix. In a few days more, this red spot becomes a conoid protuberance, the base of which is of a transparent grey colour: this cone is composed of a transparent homogeneous matter, resembling coagulable lymph, covered with a thin membrane. The cone continues to elongate, and the red point gradually disappears; the usual time being about twelve days from the period of cicatrization, but it is sooner or later, according to the extent of the joint to be restored. If only a very small portion has been removed, the red spot disappears much sooner, and the elevation is scarcely perceptible. When the growth of the first joint is completed, the vascular spot disappears; its base is circumscribed by a red border, and the covering at this part approaches in some measure to natural skin. For the next two or three days, the new growth ceases to increase in length, and begins to be gradually converted into the different structures of the limb. The extremity is rather enlarged and bulbous, and

* *Archives génerales de Médecine*, Septembre 1823.

† *Journal of Science*, No. xxxi.

is studded with vascular spots, which bleed on being touched, like the granulations of a wound. This is the commencement of the second joint of the extremity; and the process we have described is again repeated. This new growth forms an obtuse angle with the first joint, and takes about seven days to be completed. At this time the angle is observed to form a narrow depression, which is the incipient formation of the second articulation. Soon after the point of the new growth is observed to be flattened, from which a conoid projection arises, which is the rudiment of the foot: the formation of this last occupies about three days. After the foot has been completed for a few days, two vascular spots are seen on its extremity, from which the rudiments of the second and third toes originate; the others are afterwards successively developed. Sixty days after the process has commenced, the new growth and stump together do not exceed in length half the original limb, and the increase goes on very slowly, being seldom completed, at any time, in less than twelve months. The process of organization proceeds in the superior joints, while the lower ones are growing. When examined about the fiftieth day, the original nerve is found to terminate abruptly at the new growth; into which, however, it sends nervous filaments from its extremity, which is considerably swollen and expanded; and the limb is possessed of red vessels, which run in the course of the original trunks; muscles are also found in it, and the new limb is already used for the purposes of locomotion. The reproduction of the tail follows the same process as that of the limbs, and bears a much greater proportion to the original size of the part. The organization and increase are both performed within a much shorter time.

Some observations in comparison of the process of re-production in different animals follow:

"In reasoning on the phenomena of re-production, the comparison of this process, in the different animals endued with it, naturally presents itself to the mind as a method by which further knowledge of the process may be obtained.

"In the tadpoles of the frog and toad, the process is precisely the same as in the larva of the salamander. But, except in the re-production of the tail, which is both constant and vigorous, it is very uncertain in its extent, and strictly confined to certain states of development, the absorption of the tail distinctly marking the period when the power is entirely lost. It is, however, curious to observe to how near the moment of metamorphosis the reproduction of the tail continues.

"I have ascertained that the re-production of the tail of the lizard is effected by the same process as that of the salamander, and, I should think, from what may be collected from Reaumur's observations, it is probably the same in the crayfish and its species. In the snail I have also every reason to believe the same process is observed.

"From the very interesting experiments of my ingenious friend, Professor San Giovani, of Naples, I am authorised to conclude, that although the re-production of the earth-worm presents some specific differences, yet the general process is the same. I am entirely indebted to his liberality for the following account of it. When earth-worms are divided at the lower edge of their great ring, if they survive the injury, which they generally do three in twenty times, the separate parts gradually diminish in circumference, the wounds cicatrize, and the cicatrix, which is of a bright red colour, is surrounded by a round projecting band, *(bourrelet.)* In the anterior half, re-production goes on rapidly; for, in twenty-four days from the beginning of the experiment, four or six of its rings are already re-produced, in all about four or five lines in length. As soon as re-production commences, the round projecting band ceases to be observed. The new production is transparent, of a clear reddish colour, and of the same size and diameter as the rest of the body. The anus is perfectly formed, and the parts of the vessels, nerves, and alimentary canal, belonging to the new growth, can be observed, as well as the lateral filaments distributed by pairs to each of the new rings. The great ring, it is worthy of remark, entirely disappears during re-production. The process continues to proceed in the same manner, though with less rapidity, being apparently much influenced by the season of the year. Two hundred and thirteen days from the commencement of the experiment, the anterior halves have each re-produced twenty-five or twenty-six rings, the new growth becoming gradually redder, less transparent, and more perfectly organised. Their diameter is every where equal, as well in the new part as the old, so that the new tail continues conical, instead of being flattened as in the original.

"The process of re-production is much slower in the posterior halves. They preserve a healthy and well-nourished appearance, their anterior extremity cicatrizes and tapers very much, becoming more pointed, and appearing to assume the form of a head: not, however, until fifty-five days from the beginning of the experiment, can the new growth be distinctly perceived, when only three or four new rings are observed at the anterior extremity, their termination, the seat of the head assuming a pointed conical form, approaching nearer that of the natural head. Two hundred and thirteen days from the beginning of the experiment, the posterior halves have each re-produced five or six rings, and the head, in appearance, (for it does not appear that its real organization was determined by dissection,) only differs from the natural one in being more obtuse and less tapered.

"Thus, from the division of these worms, Professor San Giovani obtained six perfect ones, which he submitted to the inspection of the Royal Academy of Naples, in May 1815.

"We here observe, as in the salamander, the bulbous enlargement of the stump, the red vascular structure, the first growth of a transparent appearance, and its gradual transition to a more perfect structure. It is also probable that, had the observations been made more minutely, and more in relation to the process, the resemblance would have been found more exact.

"The specific differences which the case affords are the diminution in diameter of the original part, and the new growth at once of the same size; as also the early organization of the alimentary canal, the nerves, and vessels.

"The disappearance of the great ring, or *renflement,* the organ of copulation, is a fact both curious and interesting, although only in unison with the general economy of nature.

"This general resemblance, which we find in the process in the above-mentioned animals, can, however, with no reason be expected in the polypi, whose nutrition is carried on without any vascular system. This difference of structure and functions would only make the prosecution of this comparison, through this order of animals, more useful and necessary. But, whatever may be the difference of the process in the different classes of animals, it is very obvious that the power of reproduction itself, though more common in the lower animals, whose structure is most simple, is not dependent on the difference of organisation in the various classes, but can only be referred to some law of organisation peculiar to the species possessing it. This conclusion is most forcibly illustrated in the salamander and lizard, whose power of re-production is strictly confined to certain members; and in the tadpole, which loses this power immediately on arriving at its state of full development."

The first branch of this department, viz. Anatomy, continues to receive illustration from the publication of various systems of plates, executed in a manner highly creditable to the gentlemen by whom they have been undertaken;* while the general structure of the body, and that of the skin in particular, has been elucidated by the interesting lectures of Mr. CHEVALIER;† but, as we purpose to take an early opportunity of analysing this work, we abstain from borrowing from it here, as we are desirous of avoiding unnecessary repetition.

### MORBID ANATOMY AND PATHOLOGY.

Some interesting cases have been recorded, by LOBSTEIN and BRESCHET, of various ***imperfect developments of the brain and nervous system.***—It appears, from the observations of the former,‡ that mental imbecility bears no relation to the development of the ***great sympathetic nerve***, which, indeed, is completely evolved in all those cases, where the fœtus does not depart essentially from the human form. Thus, in an acephalous fœtus, with a double hair-lip, both sympathetic nerves were perfect in all their parts. The same was observed in a male fœtus, eight months old, which wanted the brain and cranium, and in which the cerebral nerves, perfectly well formed, were adherent to the dura mater investing the basis of the skull. Another male fœtus, one foot six lines in length, presented the following appearances:—In the place of the cranium was found a bag hanging backwards, which being cut into, besides a substance apparently cerebral and almost fluid, exhibited the appearance of a fungous excrescence, seven lines in length, and situated on

* CLOQUET, LIZARS, &c.

† *Lectures on the General Structure of the Human Body, and on the Anatomy and Functions of the Skin; delivered before the Royal College of Surgeons in London, in the Year* 1823. By THOMAS CHEVALIER, F.R.S. &c.—1823.

‡ *Op. cit.*

the cella turcica. There was no crista galli; the left portion of the nostrils was wanting, but the place occupied by a kind of proboscis, eight lines long and four broad, formed of nothing but cutis and cellular texture, pendulous from the root of the nose, and perforated by a canal which terminated beneath the cribrose plate of the ethmoid bone. There was no vestige of eyes, muscles, or nerves: the orbits, indeed, were present, and closed with the eyelids, but they were filled with cellular texture. The lingual branch of the inferior maxillary nerve was wanting, as was the hypoglossal; but the sympathetic nerves were larger than usual, and supplied branches from the superior cervical ganglion to the tongue and its muscles. Its distribution in other respects was not unnatural. There was no thyroid gland in this fœtus. One testicle had descended; the other remained in the abdomen.

Another acephalous fœtus is thus described:—It was fat and plump, weighing eight pounds and a half; its length eighteen inches; the diameter of the shoulders eight inches and eight lines; the circumference of the chest equalled thirteen inches, that of the abdomen eleven inches and nine lines; the diameter of the buttocks was six inches. In place of the brain was a soft tumor, covered by a thin epidermis, loosely adhering to the base of the cranium, and divided into two lobes; having the diameter of two inches and nine lines, and the height of twenty-one lines; partly formed of a reticulated structure, not unlike the corpus cavernosum, and partly of little bladders filled with bloody serum. The optic nerves, the trigemini, the glosso-pharyngeus, par vagum, and hypo-glossus, were present; but all these nerves were slender, and began from a free origin *(libera origine)* at the basis of the skull. The medulla spinalis was slender, and covered with a thick tunic, but gave out nerves of the usual size. The sympathetic was in all respects perfect.

In another female fœtus, there was but one eye, and the nostrils were wanting; the cerebellum and spinal cord were natural; but, in place of the cerebrum, was a round medullary nucleus, having an umbilicus on the upper surface, and giving off from the lower surface two slender optic nerves, united together, and running towards the orbit, where they ended in the bulb of the eye, which appeared to consist of a double globe; all the cerebral nerves were perfect, except the olfactory.

The trunk, ganglia, and principal branches, of the great sympathetic, were traced in a male fœtus destitute of brain and cranium. The base of the skull in this case was formed by the pars petrosa and a portion of the pars squamosa of the temporal bone and by the sphenoid, merely covered with hairy scalp. There was no vestige of any cerebral nerve except the optic, which appeared to arise by a slender origin from the sella turcica. There was no meatus auditorius in the temporal bone. The spinal marrow was extenuated and wasted; from the eighth dorsal vertebra to the first lumbar there was spina bifida, where the dura mater investing the medulla appeared naked. The duodenum in the left hypochondriac region was dilated into a large sac, which, terminating at the cœcum, retreated beneath the transverse colon: hence the course of the intestines was plainly interrupted. The jejunum arose by a slender origin from the mesentery. The capsulæ supra-renales were very small, and some lines distant from the kidneys. The genitals were likewise imperfectly formed.

M. Breschet has published two cases of children in whom the brain was imperfectly developed,* in addition to those he has already given to the public. One of these appears of sufficient interest, in reference to the late inquiries respecting the functions of various parts of the encephalon, to deserve a place in this compilation.—A male child, three years and a half old, was admitted into the Hospice des Enfans-Trouvées, about the end of 1822. It was in a state of idiocy, was small, weak, and imperfectly developed: *the head presented nothing remarkable either in its size or form;* the teeth were deficient, consisting only of the middle incisores and first molaris. The child was dumb, but not deaf, and expressed its sensations and desires by crying or grunting *(grognement)*. The appetite for food was very great. He could not stand, the lower limbs being deformed and very weak; the right side of the body was much more feeble than the left. Constantly lying in bed, the child was almost always in a state of drowsiness or stupor: on being roused, the eyes were found to be very sensible to light, the pupils much dilated, and the head was moved about either from side to side, or backwards and forwards. In the month of May, 1823, this idiot died of measles, and the following account is given of the examination after death:

"On opening the body, we found traces of inflammation in the tissue of the lungs and pleura; but the state of the encephalon was what principally arrested our attention. The left hemisphere was very imperfectly developed; the external part of this hemisphere, in all its length, was wanting; its place was supplied by a transparent membrane, which prevented the escape of an abundant serous fluid, which occupied the lateral ventricle. The incision of the membrane gave vent to this liquid, when the walls were found to be composed of two folds of the arachnoid; that is to say, of the part which covers the hemispheres, and the duplicature which lines the ventricles.

Between these two laminæ were distributed many capillary vessels, and towards the extremities of the hemisphere the liquid was of a viscid and albuminous appearance; and a little further on we perceived the cerebral substance, which was soft and white, but without any other sign of alteration. The rest of this hemisphere and that of the left side, *the middle parts of the brain,* the annular protuberance, the anterior prolongations as well as the posterior, the bulb, and the spinal cord, presented a regular development: there was, therefore, only the external part of the left hemisphere, the corpus striatum and thalamus of the same side, which were imperfectly developed. The nerves of the trunk and the limbs were dissected and compared: if there was any difference in size, it was in favour of those of the right side. The viscera of the thorax and the abdomen presented only some traces of inflammation, sequelæ or complications of the exanthema, of which the child had died."

---

* *Note sur des Enfans nouveau-nés, chez lesquelles l'Encephale offrait un Developpement imparfait. (Journal de Physiologie,* Juillet.)

We may remark, *en passant,* that the author concludes, from his observations, that, when any deficiency exists in the development of the fœtus, it is generally to be found in the median line; and that, when unequal development takes place, the defect is generally in the left side. This latter view is not confined to monstrous productions only, but is applied to the natural growth of the body; and hence we use the right arm, &c. not from any social habit, but from its more perfect and vigorous formation.

*Softening of the brain,* to greater or less extent, is a phenomenon well known to morbid anatomists, and by no means of very rare occurrence; but the attention of pathologists has lately been especially directed to the subject in a work by M. ROSTAN.* The softening of the brain is described by him as varying in degree, in its situation, and in its colour. With respect to degree, it is sometimes as thin as "boullie;" various stages intervening between this and the natural structure, although the middle between these extremes is the most common state. When the softening is inconsiderable, it becomes difficult to detect it, unless it be likewise changed in colour. The situation of this morbid degeneration may either be on the superficies or it may be deep-seated: when the seat is superficial, the convolutions of the brain are disfigured, either throughout the whole extent of the hemisphere or in a limited portion. Sometimes the softening is indicated by a change of colour in the cortical matter, which, instead of being of a yellowish grey, becomes of a rose-colour: such parts are found, when examined, to be softer than natural; if cut into, the edges are rounded; and, if the back of the knife or the handle be scraped along the surface, a part of the softened brain adheres to it. This alteration of structure generally follows the convolutions, for the most part occupying two or three inches, but sometimes extended over half, or even the whole, of the hemisphere. When more deeply seated, the corpora striata and thalami of the optic nerves are the parts most liable to be affected, and after them the centre of the hemisphere, particularly on the right side. The cerebellum likewise, and even the spinal marrow, become involved. When thus deeply seated, it is difficult to fix the limits of the diseased portion with precision; the greatest softening being in the centre, and gradually decreasing till it is insensibly lost in the natural structure. For the most part, there is but one such lesion; sometimes, however, both hemispheres are affected, and under these circumstances the softening of one exceeds, and seems to have preceded, that of the other. This morbid condition may be associated with any other degeneration, and is frequently found along with effusion of blood; which, indeed, it often surrounds. The colour of the softened portions varies: it may be yellow, green, red, like wine-leys, or a dirty white; and these colours may be found in different proportions and combinations, in the same individual. The greenish yellow is found in the centre of softened parts, which have fol-

* *Recherches sur le Ramollessemcnt du Cerveau; ouvrage dans lequel on s'efforce de distinguer les divers Affections de ce Viscère par des Signes caracteristiques.* Par LEON ROSTAN, &c. &c.—Paris, 1823.

lowed attacks of apoplexy; the rose, or red colour, exists in cases of primary softening, and is most commonly found at or near the surface of the convolutions; the appearance resembling wine-leys is not unfrequent, and resembles a spot of ecchymosis; when white, the parts are of a dull hue, like milk, and serve to heighten the pure white of the surrounding medullary matter.

The symptoms characterising this morbid softening of the brain are divided into two classes, or stages. Those marking the first stage, being common to many diseases, are uncertain. They consist in severe pain of the head, lasting sometimes for several months, but which is not constant; diminution of the mental faculties, altered manner, hypochondriasis, drowsiness, numbness of the limbs, sometimes with stiffness and contraction; the sensibility sometimes impaired, and at others morbidly increased: finally, there is occasionally delirium with great agitation, ending in mental alienation or imbecility. Perversion of sight, or even blindness, frequently supervenes, and sometimes deafness: the other senses are but little affected. Various functions are often considerably deranged: the appetite becomes impaired, the thirst increased, the tongue white, and the digestion disturbed; the epigastrium, and sometimes the rest of the abdomen, is tender on pressure; constipation of the bowels is frequent,—sometimes, on the other hand, they are purged. The stools are seldom passed involuntarily during this period of the disease, but throughout the urine is retained with difficulty. The pulse varies very much, but is seldom increased in frequency; sometimes it becomes slower than natural. Not unfrequently some violent inflammatory attack precedes the disease of the brain, and occasionally a general inflammatory diathesis. After these symptoms have continued for a longer or shorter period, the patient suddenly, or gradually, loses the use of one or more of his limbs, or of one half the body; sometimes he is comatose, but more frequently the understanding remains perfect. When the attack comes on suddenly, the patient generally recovers the use of his intellects on the next day; but fresh symptoms quickly come on, and he dies between the fourth and the fifteenth day, presenting the appearance of typhus fever.

Much rigidity of the limbs, occasionally with great pain on being touched, frequently prevails; convulsions seldom occur. The face may either be flushed or pale; and pain in the head now comes on, even if it had not existed during the first period; or, if it had formerly existed, it now becomes aggravated. The patient is generally able, after a little hesitation, to place the hand upon some particular part of the head, on being asked to do so; and this is almost always the seat of the diseased softening, and opposite to the paralysed side.

Delirium is not a frequent occurrence, but coma, more or less complete, is very common. The head is sometimes drawn backwards, with the eyes directed upwards and fixed; occasionally one pupil is more dilated than the other. The hearing, taste, and smell, are generally diminished; the mouth becomes distorted towards the latter stages of the disease, but not sooner. The symptoms have a tendency to go on progressively increasing, but the period of their duration before the fatal termination varies considerably, the disease being sometimes acute, and at others chronic. When the symptoms proceed from its commencement with rapidity, a speedy termination may be anticipated; but, if their progress be slow, the course of the disease may be very much protracted: the progress, however, is not regular, but presents various anomalies. It must be confessed, that the description given by M. Rostan of the symptoms appears to us most unsatisfactory; for, after a minute detail of appearances which may be present or absent, we are at last told that in some cases there are no symptoms whatever.

With respect to the absolute duration of the disease, little information can be gathered from the work of M. Rostan: sometimes it is asserted to run its course in a few days, and at others to require some years. In point of frequency, it is conjectured to be the most common lesion of the brain,—more frequent even than apoplexy. No method of treatment has succeeded in curing this affection, when it has attained its second period; but recovery appears to have taken place from the first stage.

The *spinal marrow* likewise becomes affected with this softening: owing to the narrowness of this cord, the whole is involved in the disease, and the symptoms are displayed alike in both sides of the body: these depend, in a great measure, on the seat of the diseased portion.

A late Number of the Journal de Physiologie contains valuable *remarks on the cerebellum and other parts of the encephalon,* by M. Serres. We hope to be soon able to lay the opinions of this pathologist before our readers in a more satisfactory manner than is possible at present, by analysing, as soon as it appears, a work on the Brain, which he now has in the press.

Three cases of *aneurism of the aorta*, from the pen of Mr. Ward, are of considerable interest, as they tend to show how far this disease is capable of assuming the characters of cynanche laryngea in its chronic form. The wheezing caused by the pressure of the aneurismal tumor has been pointed out before, but the observations of Mr. Ward are calculated to direct the attention to this resemblance between the diseases in a more especial manner. The same gentleman has related a case in which a tumor, of a soft pulpy consistence and yellowish colour, completely obstructed the circulation through the vena portæ.*

A singular case, in which a preternatural growth was found lining the inner membrane of some of the great vessels, has been related by Dr. Yelloly:† it will be found in our analysis of the work in which it appeared. To this we likewise refer for some account of an interesting paper by Dr. Davis, in which he endeavours to show that the proximate cause of *phlegmasia dolens* consists in violent inflammation of one or more of the great veins within or near the pelvis, producing thickening, the formation of false membrane, and other changes, by which the calibre of the vessel becomes too much diminished to admit of its circulating the blood in due quantity.

---

* *London Med. Repository*, October and November.

† *Medico-Chirurgical Transactions*, vol. xii. part ii.

Two cases of *rupture of the vena cava* have been recorded by Dr. Kennedy, of Glasgow, who has given references to various examples of a similar nature among the older writers.* In the first case, the injury was produced by a fall of " more than twenty feet from the top of a hay-stack." On examining the body ten hours after death, a large mass of clotted purplish blood was found in the right cavity of the thorax: " it had made its escape through a ragged longitudinal orifice, measuring six lines, in the pleural side of the vena cava superior, near the point where this vessel usually receives the azygous vein, before entering the capsule of the heart. About an inch above its termination in the right auricle, and within the pericardium, was a contraction of the vena cava, with a thickening of its coats, by which it lost more than one-third of its natural calibre." No azygous vein, nor any thing corresponding to it, could be found in this subject.

The second example occurred in a woman, who was seized with an acute malady six days after her accouchement, exhibiting the appearances of phlegmasia dolens. On former occasions, this person had passed through the various stages of child-bearing and suckling in the most favourable manner; but now referred her chief sufferings to an intense lancinating pain, deeply seated in the abdomen. Repeated venesection, purgatives, and other remedies of a similar nature, were employed without effect, and she died on the fifteenth day of her illness, within half an hour after attempting to turn in bed; at which time she uttered a faint cry, and became more and more feeble till she expired. Among other appearances, " a perpendicular rupture, with irregular edges, and measuring six lines in length, was discovered in the abdominal vena cava, between the first and second joints of the lumbar spine, and near the origin of the chyliferous duct."

From the pathology of the sanguiferous system, we naturally pass to that of the circulating mass.—MM. Prevost and Dumas have clearly ascertained the *existence of urea in the blood of various animals*, in whom the kidneys had been extirpated;† a fact from which some important inferences, both physiological and pathological, may be deduced. The formation of urea is thus found to be independent of the action of the kidneys, which would therefore seem to be merely eliminating surfaces, similar to the skin,—an opinion formerly advanced by Dr. Rollo. It is not yet made out where urea and the other ingredients of urine are formed: possibly, the examination of urine under different pathological circumstances, may tend to throw some light upon this subject. The functions of the liver, for example, seem in some degree connected with its formation, as it is well known that the urine of patients labouring under chronic hepatitis contains little, if any, urea.

The experiments of MM. Prevost and Dumas lead them to believe that the urea is eliminated by the kidneys in proportion as it is formed, and that, when these organs are extirpated, the whole of the urea is retained in the blood. If we suppose the same thing of the saccharine matter, it may easily be imagined that, where the kidneys act properly,

---

* *London Med. Repository*, October.

† *Annales de Chimie et de Physique.*

the whole of this substance will be thrown off by the blood; but, where they perform their functions less perfectly, some of the sugar will be retained,—as in cases of diabetes. This cannot, of course, be expected in any considerable quantity, as long as the kidneys perform their secretion, although but in an imperfect manner. The ingenious physiologists above mentioned seem to be of opinion, that it is thus with the sugar of diabetic patients, as with urea; and, further, that this principle possesses diuretic properties, on which the symptoms of the disease in a great measure depend. The discovery likewise serves to illustrate some of the phenomena of gout: the presence, for example, of lithate of soda in the joints of gouty individuals, naturally leads to the conclusion that this salt existed in the blood. It is known that, when the paroxysm affects the kidneys, the urine is loaded with lithic acid, and that the joints which are briskly attacked are the only ones which contain these concretions. If it were proved by analysis that, at the commencement of an attack, the blood contains a larger proportion of lithic acid than the kidneys can eliminate, according to MM. Prevost and Dumas, we would recognise the result of this morbid action of the blood in the general disturbance which constitutes the first stage of the disease; and, in the part affected, we would see a temporary seat of the secretion. These investigations are of great interest, and physiologists, anxious of repeating the experiments, will not find much difficulty in doing this. Five ounces of blood from a dog which had been deprived of the kidneys for two days, gave more than a scruple of urea; and two ounces of the blood of a cat, under similar circumstances, afforded ten grains.

In the second part of the Transactions of the Royal Society for 1823, we have some observations by Dr. DAVY on *pneumo-thorax, and the power of various textures in absorbing gases,* which cannot fail to be read with interest.

A soldier, aged thirty, was admitted into the Military Hospital at Chatham last January, labouring under symptoms of phthisis pulmonalis: he died on the 11th of February, and the body was inspected fourteen hours after. The right side of the chest appeared fuller than natural, and emitted a hollow sound when struck; on opening the abdomen, the diaphragm was found protruding into the right hypochondrium, being convex instead of concave. The body was now put under water, and a small opening made into that part of the right pleura which allowed of the readiest transit of the air. Of this, 212 cubic inches were collected in receivers, and about thirteen escaped, making in all the extraordinary quantity of 225 cubic inches. The body being replaced on the table, the thorax was examined; the water which had rushed in to supply the place of the air being carefully preserved. The inner surface of the pleura was covered with a thin layer of coagulable lymph; the right lung was very much compressed, and was joined to some of the neighbouring parts by strong bands. On inflating the lungs with a double bellows, the right lung became much expanded, the air passing freely from it into the pleura, through an ulcerated opening in the superior lobe. Here a vomica was found, which communicated with the trachea by a large bronchial tube, the extremity of which presented opposite the openings by which the cavity communicated with the bag of the pleura. At the point of communication between the lung and chest was discovered a vascular apparatus, which was formed in this manner: "Between the false membrane of the vomica and the pleura there was a small irregular sinus, not exceeding an inch in diameter, the sides of which, though not adhering, of course were in contact, or very nearly so. This sinus was the channel of communication, and contained the vascular structure alluded to. It opened into the cavity of the chest by a hole in the pleura pulmonalis, about the size of a crow-quill, and into the vomica by three smaller holes in the substance of the lung, not corresponding with the former, so that a probe could not be passed from one into the other in a straight line; and, consequently, when the surfaces of the sinus were pressed together by the compression of the air in the pleura, in the act of expiration; the communication through which the air entered was closed, and its exit prevented."

Besides this large vomica, various smaller tubercles were contained in the right lung, particularly in the superior lobe: these were of different sizes and degrees of opacity; all were solid, and none had suppurated. The left lung likewise contained small tubercles, but was free from any adhesions. The pericardium contained three ounces of water, and the ventricles of the brain had more fluid in them than is usual. No air was found either in the blood-vessels or cellular membrane, in any part of the body.

The water which had been removed from the chest was turbid, and appeared to contain pus; the most important part of the history, however, relates to the air which was found in that cavity. This was found, by chemical examination, to be composed of eight of carbonic acid gas, and ninety-two of azotic, in the hundred parts. The history of the case, as given above, can leave little doubt of the air found in the chest being atmospheric air altered; and the question to be determined is, what had become of the oxygen, which had disappeared, and whence the carbonic acid gas? To determine the former of these points, the following experiment was instituted:

Atmospheric air was injected into the pleura of a dog, and the opening through the parietes of the chest closed by suture: at the end of forty-eight hours the animal was killed. An hour after, the pleura was opened, and about eight cubic inches of air collected, which showed slight traces of carbonic acid gas, and consisted of ninety-three of oxygen, and seven of azotic gas, in the hundred. "The result, then, of this experiment (says Dr. Davy,) seems to show that the oxygen was absorbed in a greater proportion than the azote, and thus tends to account for the accumulation of the latter gas in the preceding case." With regard to the carbonic acid gas, it is conceived to have been formed in the lungs as in ordinary respiration, and, mixing with the air inspired, was thus received into the pleura: if so, it would seem to prove that this gas was less readily absorbed by the pleura than oxygen. To ascertain this, we have the following experiment:

A bladder was filled with about thirty cubic inches of air, consisting of atmospheric air eighty parts, and carbonic acid gas twenty parts: at one end of the bladder was a stop-cock, and at the other a trochar. The trochar was thrust through the integuments of the chest on the right side of a dog; the stillet was withdrawn into the bladder, upon which the air rushed through the canula into the thorax, being partly forced back again on expiration. The quantity retained, however, is supposed to have exceeded ten cubic inches. The canula was withdrawn as speedily as possible, and the wound closed as before. The health of the animal appeared to suffer but little. The operation was repeated, two days after, on the other side of the chest, and a mixture of seventy-five parts of common air with twenty-five of carbonic acid gas was introduced. At the end of twenty-four hours the dog was killed, having apparently suffered little from either experiment: the result of the examination is thus stated. "About three cubic inches of air only were procured from the left* pleura, which were found to consist of 18.3 carbonic acid gas, 78.3 azotic gas, 3.4 oxygen gas; whilst the air admitted consisted of 20.0 carbonic acid gas, 63.2 azotic gas, 16.8 oxygen gas: thus apparently showing that, during a sojourn of three days in the pleura, the oxygen had been absorbed in a greater degree than the carbonic acid gas, and the latter in a greater degree than the azote. The result of the experiment on the left pleura was very similar: it afforded ten cubic inches of gas, consisting of 25 carbonic acid gas, 70.6 azotic gas, and 4.4 oxygen gas. The appearances, on examining the wounds, were satisfactory: the cavity of the chest was free from inflammation, the lungs uninjured, and the cicatrix in the pleura only just perceptible."

The result of these experiments justifies the opinion of the author, that the carbonic acid gas was not formed by secretion from the pleura, but, on the contrary, it would appear that this membrane has the property of absorbing gases, exhibiting a preference for one over another; and the discovery of this circumstance led Dr. Davy to pursue the subject farther.

Three other gases were injected into the pleuræ of dogs,—viz. hydrogen, nitrous oxide, and nitrous gas. "About twenty cubic inches of a mixture, consisting of 57.5 parts of carbonic acid gas and 42.5 of hydrogen, were admitted into the left pleura of a dog, in the manner and with the precaution already noticed. The health of the animal was not apparently affected. At the end of two days, about thirty cubic inches of a mixture, consisting of 44.5 of azote and 55.5 of nitrous gas, were passed into the right pleura. Immediately the dog's breathing became quick and short, but not laborious; it refused to eat, and expired in the evening, at the end of five hours from the time that the air was introduced. The next morning the body was examined. About six cubic inches of air were collected from the left pleura, consisting apparently of 12 carbonic acid gas, and 88 azote. After the carbonic acid gas was removed by lime-water, the residual gas extinguished flame, and was not itself in the least inflammable; whence the inference is that it was azote, or at least principally azote, as the presence of a small quantity of hydrogen might be concealed, and escape detection. From the right pleura, about five cubic inches of air were

* This ought evidently to be *right*.

procured, which consisted of 6.9 nitrous gas, or air absorbable by a solution of green sulphate of iron, and of 93.1 azote. On opening the chest, the wounds in the pleura were found closed; the pleuræ were of natural appearance; the substance of the left lung was redder than usual,—that of the right was dark-red, and it contained a good deal of blood and serum; the bronchia did not exhibit decided marks of inflammation; the right auricle and ventricle, and the venæ cavæ, were distended with grumous blood, and the left auricle and ventricle, and aorta, contained a good deal of liquid blood, which, as well as that of the venous system, had lost its peculiar tint, and acquired a chocolate hue.

"The obvious results of these two experiments in the same dog, are, 1st, the absorption of the greater part of the carbonic acid gas, and the whole of the hydrogen, introduced into the pleura, and the appearance *de novo* of a considerable quantity of azote; 2dly, the death of the animal in the space of five hours from the time of admission of the nitrous gas and azote into the opposite pleura; the absorption of the greater part of the former gas, without inflammation of the membrane with which it was immediately in contact; and the production of a peculiar change in the blood."

The author has twice repeated the experiment of introducing hydrogen into the chest, and in every instance has found this absorbed, and its place supplied by a small quantity of azote. That the azote did not previously exist in the pleura, as some have conjectured, was proved by opening the thorax of dogs under water, when not a bubble of air escaped. Dr. Davy inclines to the opinion that the azote is formed from the blood, in the way of exhalation or secretion, and alludes, in further illustration, to the well-known fact of air being frequently found in the blood-vessels after death, under circumstances which preclude the possibility of its arising from incipient decomposition, or from foreign introduction from any vessel wounded during the dissection.

The nitrous gas appears to produce its deleterious consequences after it has been absorbed, as it does not excite inflammation of the parts with which it comes into contact.

In an Appendix to this paper, a very interesting case is given, in which pneumo-thorax was discovered during life, and the symptoms relieved by puncturing the chest. A soldier was sent home from the West Indies, invalided in consequence of hæmoptysis, which had supervened upon a severe fall, by which he had received an injury on the left side of the chest, about eighteen months before. He was admitted into the Military Hospital at Chatham, on the 9th of May, 1823, and his case presented nothing remarkable till the 13th; early in the morning of which day, after a violent fit of coughing, symptoms of pneumothorax came on suddenly, and continued increasing till the 21st. "The most prominent symptoms were, a feeling of extreme tightness about the chest and abdomen; rapid and difficult inspirations, between thirty and forty in a minute; great anxiety of countenance and agitation of mind, accompanied with a small pulse of 130; cold sweats frequently breaking out on the neck and face; and a considerable prostration of strength. On examining the chest, the left side was found more protuberant, and in all its dimensions larger, than the right; it was tense, and, on percussion, sounded remarkably hollow and tympanetic, giving the idea of its being distended with air; and the heart was found beating on the right side, under the mamilla."

Under these circumstances it was resolved, on consultation, to tap the chest; which was accordingly done, in the following manner:—A trochar was attached to an empty bladder, in the manner formerly described, and the parietes of the chest punctured between the eighth and ninth ribs, the integuments and intercostal muscles being previously divided with a scalpel. A little air only rushed out, (not exceeding five cubic inches,) and, as it was concluded, from the symptoms continuing, that its escape had been prevented by adhesions of the pleura at the point which had been perforated, the operation was repeated next day. The puncture was now made just below the left papilla, when, on withdrawing the stillet into the bladder, a large quantity of air rushed out, and distended it: the bladder was now separated, having secured its contents by the application of a ligature. Air continued to rush from the chest for several seconds, "as if from a blow-pipe." When this ceased, and the air began to pass inwards during inspiration, the canula was withdrawn, and the wound healed.

The relief to the patient was great and sudden, and he had continued to improve up to the 17th of June, on which day the paper is dated. At this time his appetite was good, his cough but little troublesome; he could lie on the left side, which he had been prevented from doing for many months previously; the wounds were healed, and the chest considerably diminished in volume, being also much less tense and tympanetic. The heart, however, continued to be felt on the right side, and the fluctuation of a fluid was perceptible in the left: nevertheless, Dr. Davy seemed to hope he might eventually recover.

The air collected in the bladder was now examined, and found to consist of 93 parts of azotic gas, and 7 of carbonic acid; being almost the same with that found in the fatal case above mentioned. The source of the accumulation of air in the chest was likewise probably analogous, particularly as it supervened upon a violent fit of coughing.

The same paper contains some very interesting remarks on the *secretion of air* in the human body,* and its effusion into closed cavities. In addition to the illustration afforded by the cases which we have detailed, two others are given, in which, very shortly after death, and before there was the least appearance of putrefaction having begun, Dr. Davy detected air in the body, and collected it in sufficient quantity for examination. In the body of a soldier, aged twenty-seven, who had died of chronic dysentery, with an ulcer of the larynx, the cellular membrane in both mediastina was vesicular: the vesicles were broken under water, and half a cubic inch of air collected; this consisted of oxygen 7, carbonic acid gas 4, and azote 89. The oxygen is conjectured to have been extraneous, being derived either from atmospheric air adherent to the vesicles, or having penetrated them during half an hour that they remained exposed.—In another instance, air was collected from vesicles

---

* See some interesting observations on the formation of gases in the human body, by Dr. GRANVILLE, in the Number of this Journal for February, 1822.

on the surface of the lungs; this consisted of azote 5 parts, and 1 part of carbonic acid. Some experiments were made with a view of ascertaining whether mucous surfaces be capable of absorbing air, and the result rather inclined Dr. Davy to suppose they are. It must, however, be acknowledged that these experiments are extremely inconclusive.

Very little information of novelty or importance has fallen under our notice with respect to the *digestive organs,* during the last six months: perhaps, the most interesting pathological facts in this department are two cases of *rupture of the liver,* detailed in the pages of a respected contemporary Journal.*

### THERAPEUTICS AND MATERIA MEDICA.

We find very little on which to congratulate ourselves in this department: the means of cure necessarily follow the other branches of medical science, with slow and unequal steps; and we know the pathology of many diseases, in the treatment of which we are lamentably deficient.

Dr. Heller has made a series of experiments on *prussic acid,** the results of which are very different from those of his countryman, Magendie, having entirely failed in his attempts to cure pulmonary consumption by its means; notwithstanding that he has carried the dose to the extent of fifty or sixty drops of the medicated acid, (that is to say, from twelve to fifteen drops of the pure acid,) in twenty-four hours. In asthma and hooping-cough he has found it of service: with regard to this last complaint, his experience seems to justify the commendations bestowed upon it by Dr. Granville. Dr. Heller's words are—"I effected more in hooping-cough than in all the other diseases of the chest of which I have yet spoken; that is to say, a cure, which (if I may be allowed the expression) was complete." He likewise regards it as efficacious in hæmoptysis: but the most remarkable and important of his experiments upon this subject relate to its employment in diseases of the heart. He informs us, that he was induced to undertake these from observing the facility with which prussic acid abolished the contraction of the heart, in animals which were submitted to experiment. He states that he has met with six examples of persons affected with aneurism of the heart, in whom he has been enabled, by means of this acid, to diminish the force of the palpitations, by weakening the contractions, and by moderating the flow of blood towards the organ. Taken, at first, in doses of ten drops in twenty-four hours, this medicine has been carried to the extent of above sixty drops, not only without inconvenience, but with the effect of producing a marked improvement, after other remedies had failed. In three of the patients, the aneurism had already existed for many years, and had acquired a force and power which subjected the life of the patient to constant danger; the medicine retarded but little the fatal termination, but it had the great advantage of moderating the action of the circulating system, and thus gave relief to the respiration. It appears, from the statement of Dr. Heller, that

* *London Medical Repository,* August.

† *Revue Medicale,* August and September.

these aneurisms had been previously treated by means of general and local bleeding, severe regimen, digitalis, refrigerants, blisters, and all the other remedies usually had recourse to on such occasions; but without success, and even without affording any relief.

But the result was still more favourable in three others affected with aneurism, to whom the prussic acid was administered: these are still patients of the author's, and he has the satisfaction, from time to time, of moderating the action of the heart by the exhibition of this medicine. Among them is a man of science, who at first manifested great reluctance to take the prussic acid, being aware of its deleterious nature, but he himself now regulates the dose of the acid which he takes in twenty-four hours, by the force of the heart's action: thus, his pulse, which beats at the rate of 116 in the minute when he remains some days without taking the medicine, only beats at the rate of 98 when he has taken it forty-eight hours, and falls to 80 when he has continued its use for several successive days. It is of importance to remark, that the effects of the remedy, according to Dr. Heller, are not perceptible in the commencement of the treatment, and that it is only when the dose has arrived at thirty or forty drops in the day, that the abatement of the circulation is observed. He recommends that we should not exceed ten drops a-day to begin with, and this is afterwards to be increased by five drops at a time.

In epilepsy, the medicine seemed to have the effect of postponing the fit, and sometimes of rendering it less violent. In hypochondriasis, it entirely failed.

Some apparent advantage was gained from it in hydrophobia affecting dogs and horses; and hence Dr. Heller is led to entertain favourable anticipations of its powers in its disease. These speculations derive no support from the case which recently occurred in St. George's Hospital, in which the prussic acid was pushed as far as was consistent with the safety of the patient.

Various cases of hysteria are given, in which this medicine was employed with advantage; and, among others, the following instance of nervous palpitation of the heart is related.—A young lady, aged twenty, who had been married for two years, had been for six months affected with violent chagrin, originating in a passion which it became her duty to overcome. For two months the respiration had been difficult, with frequent tremors, constant oppression, and an accelerated pulse,—symptoms to which she paid no great attention; when, after a short time, the heart itself became the seat of an affection, which consisted of violent nervous palpitations. These re-appeared several times a-day, lasted but a short time, and left pretty long intervals; but, on the slightest lively impression, they returned with much force, and were extremely disagreeable to the patient. In this state she took the prussic acid, without any other treatment, and had to congratulate herself on its employment; for she had not exceeded the dose of thirty drops a-day, when the palpitations were already much diminished, the respiration having become easier, and her improvement very marked. At the end of some days she discontinued the acid; but the palpitations returned, and she was consequently obliged to resume the use of the medicine.

This time she continued it during five weeks, without interruption; by which means she got entirely rid of the palpitations. It has likewise been tried by Dr. Heller in various other diseases, with results more or less satisfactory; and its external application is recommended in tic douloureux, chronic rheumatism, and dartres.

The *sulphate of quinina* has been recently tried by various practitioners in this country, among whom Dr. ELLIOTSON, of London,* and Dr. DICKSON, of Clifton,† have published the results of their experiments. Of the former, an account will be found in our analysis of the volume in which they were contained. Dr. Dickson gives three cases of intermittent, in which this remedy proved efficacious. His paper contains a short extract from a letter of Mr. BRODIE's, in which he states that he has seen the sulphate of quinina exhibited several times, and believes it to be a very useful addition to the Pharmacopœia.

M. ANDRAL, *fils*, has lately made some experiments, at La Charité, with *strychnina* and *brucina*, from which he draws the following conclusions:—1st. Pure strychnina acts upon man in the same manner as the extract of nux vomica, but with a much greater degree of intensity. 2d. The action of the strychnina is so energetic, that it ought only to be employed with very great precaution. Its effects vary in a remarkable manner, according to the susceptibility of the individual. Thus, in one, a twelfth part of a grain was sufficient to cause serious inconvenience, whilst, in another, the dose of strychnina could be varied, almost with impunity, to somewhat more than a grain. 3d. Brucina acts on man as on other animals, much less energetic than strychnina, since we may, without inconvenience, commence its use in doses of half a grain. It may be substituted with advantage in medicine for the alkali of nux vomica. 4th. Considered with regard to their therapeutic properties, strychnina and brucina show themselves more or less efficacious, according to the kind of palsy which is attempted to be cured with them. Employed in cases where the paralysis is associated with an inflammatory state of the brain or spinal marrow, they will very probably aggravate the symptoms. In individuals who remain hemiplegic after an hemorrhage in the brain, the use of these alkalies is, for the most part, unavailing; and it is even to be apprehended that they may excite inflammation of the cerebral substance round the site of the apoplectic effusion. But there are cases in which the palsy seems to remain after the re-absorption of the effusion, by a sort of habit; such palsy may yield to the alkalies of the nux vomica and spurious angustura. Finally, these same alkalies appear particularly efficacious in those forms of paralysis, the causes of which cannot be referred to any lesion of the cerebral centres; such is the kind of palsy to which individuals working in preparations of lead are especially subject. The preceding observations show the efficacy of strychnina and brucina in this species of palsy; out of nine persons affected with it, six were cured, or at least relieved.

---

* *Medico-Chirurgical Transactions*, vol. xii. part ii.

† *Edinburgh Med. and Surg. Journal*, October 1823.

M. Andral has seen other cases of palsy of the same kind, which have likewise yielded to the alcoholic extract of nux vomica.*

We are indebted to MM. Prevost and Dumas for one of the most novel and ingenious applications of chemistry to therapeutic purposes, which is to be found in the records of the healing art.† This consists in the application of ***galvanism to effect the decomposition of urinary calculi within the bladder.*** Whether this process shall ever be so modified, and so much under control, as to admit of its use in the human subject, is matter of doubt; but the subject is unquestionably worthy of attention in the present age of discovery. Did not the functions of the parts require to be carefully preserved, it is no doubt true that, by a battery of sufficient intensity, a calculus, composed of alkaline or earthy salts, might be extracted from the bladder by the simple introduction of a double sound, communicating on one hand with the calculus, and on the other with two vessels filled with water, in which is plunged the opposite poles of a galvanic apparatus. This arrangement would draw the acids of the calculus into one vessel, and the bases into the other; but, so far as the experiments have hitherto been carried, this degree of galvanic operation excites too much irritation in the bladder to be admissible. Another resource yet remains, however, apparently of a more practicable nature. This consists in giving to the calculus a tendency to crumble from the slightest force; such a friability, in short, as shall render it easily broken into pieces sufficiently small to be evacuated by the urethra. A fusible calculus from the human subject was submitted to the action of a pile, consisting of 120 pairs of plates, for twelve hours in succession. The platinum wires constituting the poles were placed in contact with the calculus, about six or eight lines distant from each other, and the whole plunged into a vessel filled with pure water. During the galvanic action, the bases and the phosphoric acid first arrived at their respective poles, then entered anew into combination, and the salt thus formed again was precipitated in the form of powder. The calculus weighed ninety-two grains before the experiment, and was reduced at its termination to eighty. The process being continued, at the end of sixteen hours it presented a mass of such friable texture, as to be reduced into small chrystalline particles by the slightest pressure; the largest of these not exceeding the size of a lentil, and consequently quite capable of passing through the urethra.

In order to ascertain how far this decomposition could be effected in a living body, MM. Prevost and Dumas, selected a dog, of rather large size, into whose bladder they introduced a fusible calculus attached to a sound, and between two conductors of platinum; the bladder was next distended by injecting tepid water, and the apparatus subjected to galvanic influence. After a little struggling, the animal became calm, and was subjected to the operation during an hour. On removing the sound at the end of this time, the calculus showed unequivocal signs of decomposition. The same process was repeated night and morning

---

* *Journal de Physiologie*, Juillet. † *Idem.*

during six days, when the friability of the calculus rendered it impossible to continue the experiment. It had lost weight in the same proportion as in the preceding example. The bladder, which was examined by killing the animal a few days after, exhibited no appearance of disease. The ingenious authors assure us, that the bladder suffers no inconvenience from this more moderate degree of galvanic operation, and suggest, as a proof of the mildness of its influence, that we should immerse the tongue in a vessel filled with water, in which a calculus is undergoing decomposition, after the manner described in the first experiment. We are informed that the tongue (an organ more sensible than the bladder,) scarcely perceives the galvanic action, even when the decomposition is going on very briskly.

"In reflecting on these facts, (say the authors,) it is perhaps admissible to hope that, with suitable modifications and appropriate apparatus, this principle may be applied to the extraction of calculi formed by saline combinations ; but it is quite evident that it can offer no advantage for the extraction of those which only consist of uric acid, or which contain a large proportion of this with relation to the other constituents. But, before even thinking of trying this application, we wish to institute a more thorough investigation of the following points: 1st. We have introduced calculi into the bladder through an opening made in this organ at its anterior part, and we intend to operate upon these animals in various ways, with a view of ascertaining accurately that which ought to be preferred for the human subject. 2d. It is desirable to establish, by a series of experiments, what the liquids are which answer best as injections into the bladder : we imagine that pure water, as used by us, is probably not the most advantageous. 3d. It becomes indispensible to ascertain the nature of the calculus before-hand, that patients may not be exposed to painful attempts, which may be fruitless." In a note appended to this paper, (probably by MAGENDIE,) we are informed that these experiments have been repeated, in Paris, upon animals at the Jardin des Plantes, with similar results. A certain quantity (the proportion is not mentioned,) of nitrate of potass to the water injected into the bladder, is said to expedite, and otherwise facilitate, the decomposition.

At page 35 will be found an interesting case of pneumo-thorax, in which *tapping the chest* was practised with success; and in the surgical department, some remarks on the various therapeutic applications of *acupuncturation*.

---

### SURGERY.

The last six months have been remarkably barren in the detail of great or extraordinary operations ; nor has there been any very striking novelty exhibited in this department of the art: indeed, every year renders it more difficult for us to expatiate upon the operations of surgery. It is true that new suggestions are occasionally thrown out, and new operations either proposed or attempted; but most of them are either inadmissible in themselves, or are only revivals of antiquated modes, which have fallen into disuse. The extirpation of the uterus, and the recto-vesical operation for lithotomy, may be classed among the former of these; and the *Tagliacotian operation* of forming a new nose is a specimen of the latter. This last operation has, we are informed, been lately attempted at St. Thomas's Hospital; but we are not acquainted with the details of the case, and only know from public rumour that it was not successful. We should be unwilling to say any thing that could tend to lessen the zeal, or slacken the endeavours, of those who labour towards the perfection of this important branch of our art; but we cannot think that the formation of a new nose from the skin of the forehead, for the sole purpose of decreasing deformity, is very likely to succeed. The operation is tedious and painful; the chance of forming a proboscis at all natural in its appearance, not great; and the scar on the forehead is, of itself, a great drawback to the article of personal beauty. When to this is added the probability of the part sloughing instead of uniting, we cannot help thinking that the making of new noses had better be left in the hands of the machinist, where it has hitherto been trusted, with considerable benefit to that class of sufferers.

In the Quarterly Journal of Foreign Medicine,* we find the detail of a tremendous operation for *the extirpation of the thyroid gland*: it is taken from GRAAFE and WALTHER'S Journal, and the surgeon, HEDENUS, of Berlin, says that he has operated successfully in six cases.

"The patient was a young man, twenty-one years of age, of scrofulous constitution, who had had bronchocele from his youth, and which had now attained the size of a skittle-ball, covering the whole of the fore part of the neck, from the os hyoides to the manubrium sterni, and on both sides pushed back the sterno-cleido-mastoid muscles and parts adjacent. Its circumference at the base was fourteen inches; its transverse diameter seven inches. It was firm, tense, and heavy; the surface abounding with varicose veins. It was attached firmly to the larynx, and encroached upon the trachea, exciting a distressing rattling respiration, with threatening of suffocation, especially on quick walking or ascending stairs. He was operated on in the theatre of the hospital at Berlin, in the presence of numerous spectators.

"The patient was laid on a mattress. I considered the recumbent posture as most convenient to the operator, and comfortable to the patient. I then divided the skin in a longitudinal direction, with a convex edged bistoury, from the os hyoides to the top of the sternum: not being able by this means to form any flap, I dissected back the skin and platysma myoides on both sides from above downwards, so as to form a flap of two inches, turned back on either side. I then saw the sterno hyoid and sterno thyroid muscles much increased in size, and firmly fixed to the whole tumor, and destitute of the usual aponeuroses: these I endeavoured, for the sake of sparing them in the operation, to dissect and separate from the swelling. Scarcely had I dissected away a quarter of an inch, when blood streamed in great quantity from numerous

* No. xix.

small arteries, which could neither be tied, on account of their minuteness, nor restrained by any styptic application, since their mouths were retracted and hid beneath the strong and firm aponeurosis which invested the tumor, so that they could not be reached or withdrawn. I gave up, therefore, the attempt to separate these muscles, and came immediately to the resolution of cutting them through at their points of attachment above and below, and removing the included portions with the tumor. I next (continues our author,) separated the swelling above and below, from the sterno-cleido-mastoid and omo-hyoid muscles, as also from the jugular veins and carotid arteries, which were intimately connected with the tumor by cellular membranes, until I freed them as far as the point where the thyroid arteries come off; I then tied both the superior and inferior thyroideal arteries of either side, close to the tumor, and, on account of the free anastomoses, used the double ligature, dividing the vessels in the space between. The deeper I carried my dissection now, the more serious did the operation appear, since, at every cut of four or five lines, I was obliged to tie two or three arteries; and these ligatures, on account of the size of the tumor, and the deep situation of the vessels, were very difficult, and required great care.

" When I had, with the greatest caution, dissected to the base of the tumor, which was firmly fixed to the thyroid cartilage and the three upper rings of the trachea, on every side so great a number of arteries presented themselves, for the most part of the diameter of the radial or digital arteries, that I could not cut a line or two without dividing two or three of them. Under these circumstances, and as the patient, notwithstanding the speedy ligature of the arteries, (effected partly by means of the artery forceps, partly by the needle and thread,) had already lost a very considerable quantity of blood, I hesitated not, that I might bring the operation to a more speedy close, and spare as much as possible the loss of blood, to adopt at once the method since recommended by Brunninghausen,—namely, to tie the base of the swelling, and then, with the bistoury, to extirpate the tumor above it. I used for this purpose a blunt-pointed aneurismal needle, armed with two four-threaded ligatures; and, while I directed the tumor to be pulled upward, passed the needle through the middle of its base, and, drawing the ligatures through, I tied one below, the other above, as firmly as possible. However, that I might perfectly command the bleeding, I passed also a third ligature around the whole circumference of the swelling, and extirpated the tumor now without any bleeding from the parts included in the ligature. I now cleaned the wound from blood with a sponge, that I might see if any important vessel was yet bleeding; and as this was not the case, there being only an oozing from the small vessels of the wounded surface, I placed the ligatures over the lips of the wound, and fastened them there by means of plaster. I sprinkled the whole wounded surface with powdered gum arabic, and laid on this agaric wet with Theden's vulnerary water, and, filling the remaining cavity of the wound with charpie, I brought the lips of the wound as much as possible together by adhesive plaster; over the plaster I laid dossils of lint and compresses wet with vinegar, which were renewed every six or eight minutes; the whole was secured by a bandage."

We feel compelled, by our duty as Journalists, to give this case as a novelty; and we beg leave to add, that we sincerely hope it will long continue to be so.

Dr. WEINHOLD, of Halle,* relates three cases of *the successful extirpation of schirrus parotid gland*, in opposition to the opinion of Mr. BURNS, who declares this operation to be impracticable. Dr. Weinhold says, that there are three stages of this disease,—incipient induration, confirmed schirrus, and open cancer: in the first, the gland may be extirpated with the knife; in the second, it may be destroyed by ligature and caustics; the third condition alone is incurable. In three instances, Dr. W. affirms that he has removed this gland. He does not deny that the operation is dangerous; that there is danger of wounding the carotid artery, or dividing the par vagum. We do not envy the style in which Dr. Weinhold has couched his remarks; they are neither just towards Mr. Burns, nor is there any very good taste or feeling displayed in the unnecessary allusion to the death of a late celebrated statesman in this country.

In HUFELAND's Journal, we find it proposed by M. MAYER to remove the *polypus of the nose* by means of the powder of the *marum verum*, taken as snuff, to the amount of five pinches in the day: it is productive of occasional hemorrhage, and ends by destroying the polypus. A case is given by M. Mayer, in which the tumor had grown after repeated extirpation, and which was cured by the marum taken as snuff for a short time. This proposal, unlike some of those emanating in Germany, has therefore actually succeeded; and we are the more anxious to give currency to the above fact, as the operation of extirpation is a very barbarous one, and seldom affords more than a temporary relief.

*Acupuncturation* has, within the period now under review, been resorted to as a remedy for the cure of other diseases besides rheumatism. We find, in addition to the testimonies of Dr. SUTTON and Mr. FINCH, that a patient labouring under anasarça of both the upper and lower extremities, as well as the trunk, the cellular membrane being enormously distended with fluid, accompanied with cough and most distressing dyspnœa. Dr. TWEEDALE, of Lyme Regis, who relates this case,† was induced to make trial of acupuncture, in consequence of the patient having experienced but little relief under very active treatment. It was performed with a common needle of middling size, with a thread passed a few times round it at rather less than a quarter of an inch from the point, in order that the punctures might not exceed the depth required. A dozen punctures were made within a very few minutes, with little or no pain to the patient. The result was, that at the end of a week the arms and trunk returned to their natural size, and nothing remained

* *Journal Complémentaire*, Novembre.
† *Medical Repository*, October, p. 313.

but slight œdema of the ankles and feet. As the punctures had closed, the operation was repeated with a small glover's needle, the point of which, being triangular, leaves a more permanent puncture, and also enters the skin more readily.

An application of *acupuncturation* has recently been made to the treatment of *convulsive diseases*, which at least has the merit of novelty. —A carder of wool was attacked, on the 4th January, 1822, with convulsions of the muscles of the abdomen, diaphragm, thorax, neck, and head, in consequence of a fall, in which the ensiform cartilage was violently pushed backwards. Different means were employed for three days without avail, at the end of which oppression of the lungs came on. At this time the convulsions somewhat abated, and intense pain was experienced beneath the last false rib of either side. This state lasted until the 20th of May, when M. Haime, having seen the patient along with Dr. Pipelet, the ordinary attendant, recommended the employment of the acupuncturation. He pierced with a needle the body of the right sterno-cleido-mastoid muscle, in the direction of its fibres, and pushed the needle into the depth of twenty-seven lines. The convulsions ceased immediately. He introduced another into the fleshy portion of the muscle near the sternum. (In these punctures the needle enters with difficulty, because at this part the skin is strongly attached.) The instruments remained twenty-five minutes in their place. During this time there were no convulsions, but, as soon as the needles were removed, they returned, although but slightly. The puncture of the skin caused some pain, that of the muscle gave less. Next day he applied a needle to the left side; he pierced the muscle diagonally, and brought out the needle through the skin about six lines, and guarded the point of it with soft wax; the object of which was, that it might not enter again, nor prick the neighbouring skin. The effect, with regard to the convulsions, was less than when the operation was performed on the right side. After this failure, he took away the needle from the left side, and again applied it to the right, where he left it during nine hours: the convulsions ceased. Next day he again applied the needle on the right side, when he allowed it to remain for four days. There were but very slight convulsions. He remarked, by degrees, that the muscle became swelled, the needle adhered less and less; then the convulsions returned, and gained strength in proportion as the needle became less adherent. This being removed, the swelling disappeared, either spontaneously or in consequence of the application of emollient poultices. The convulsions of the neck alone have proved obstinate; they move the head from left to right, and elevate the left side. For a year, matters have remained nearly in the same state.

Dr. Pipelet occasionally intermitted the acupuncturation for a considerable time. On these occasions, the convulsions of the neck increased, and the patient requested that the needles might be re-applied. At present they are had recourse to every ten or fifteen days, and the convulsions are observed to return if a longer interval be allowed to intervene.*

* *Journal Complémentaire*, Aout.

Another case in which acupuncturation has been resorted to with success, is also recorded in the Medical Repository: it was in the person of a man who had fallen from a considerable height, and received several extensive lacerated wounds on various parts of the body and scalp, in consequence of which trismus had taken place. The patient was in the most distressing situation, with a pulse of 130, the jaw completely locked, and, from the extreme rigidity of the muscles about the throat, he was incapable of swallowing the smallest quantity of liquid. Mr. FINCH, of Greenwich, who relates this case, introduced a needle into the masseter muscle of the right side, and was much gratified to find that muscle, as well as the sterno-cleido-mastoideus, platysma myoides, and all the muscles of the neck and throat of that side, instantaneously relieved from their spasmodic contraction. Another needle was introduced on the left side, and relief, though not to the same extent, was immediately afforded: he was enabled to swallow immediately a large dose of opium, and recovered perfectly.

The Italian surgeons still continue to practise and to praise the *temporary ligature of arteries*, in cases of aneurism. Dr. MEDORO* has published a case of this kind. The aneurism was situated in the ham, and the operation performed in the usual place in the thigh; the ligature was flat, and tied over a cylindrical compress, to which a ligature was fastened for the purpose of facilitating its removal. It was taken away in eighty hours after the operation. The firm adhesion of the artery made it impossible to raise it by the ligature, but, guided by it, Dr. MEDORO passed his finger into the wound, his nail touching the cylinder, and on it he cautiously divided the ligature with a convex-edged knife. The author thinks that, without this ligature, it would have been impossible to have withdrawn the cylinder from the wound.

We do not add any remark upon this case, having so recently given our decided opinion against this practice: it can only have the effect of hastening the healing of the wound by a few days, and this slight advantage is more than counterbalanced by the difficulties it adds to the operation.

M. VERDIER† has published an account of an apparatus, which is in fact a modification of that of Camper, for the compression of the external iliac artery in cases of aneurism. He applied it in the instance of a man, aged thirty-one, who had two aneurismal tumors on the same side, —one in the ham, the other in the groin. It is sufficient to observe, that M. Verdier's apparatus was put on in February 1817, and in the same month of 1822 we are told the tumor was *scarcely perceptible*, and the *pulsations very feeble:* however, in the spring of that year, the man died of aneurism of the aorta. Now we, for our parts, cannot conceive the advantage of a method which obliges the patient to keep his bed for five years, and at the end of that time evidently leaves the disease uncured.

---

* OMODEI's Journal.

† *Journal Universelle,* Aout.

In Italy, we find that a contest has been going on,—a very amicable one, however,—between the celebrated Professors SCARPA and VACCA, relative to the *recto-vesical operation.* We need not, we believe, enter deeply into this discussion, because we are quite sure that, in this country, the new mode of operating is not likely to gain a footing. M. Scarpa's reasons in opposition to it are, in our opinion, sound and judicious; they are principally these: first, that the vertical section of the membranous part of the urethra and prostate gland cannot be made without separating the left common seminal duct from the vas deferens and vesicula seminalis of the same side; secondly, because the fæces cannot be prevented from entering the wound, even according to the improved method employed by Sanson: in short, as he truly observes, there is too much resemblance between this operation and that by the apparatus minor, when imperfectly performed. We have read, with great attention and much pleasure, this celebrated surgeon's letter to M. Vacca on this subject, and cannot conceive that any dispassionate man can be otherwise than convinced of the impropriety of this operation, who considers the force of what M. Scarpa has therein urged.

M. CIVIALE* has published a treatise upon *strictures of the urethra and calculi in the bladder,* which we only notice on account of a proposal which he makes of extracting the stone through the urethra, which he gradually dilates so as to enable him to pass in an instrument for the purpose of laying hold of the calculus, which he then breaks with another instrument, and extracts the fragments. We have already condemned this practice. Sir ASTLEY COOPER has demonstrated the possibility of extracting small calculi even from the male bladder, which is more than could have been anticipated, and as much as ever can be attempted, in our opinion; and it is obvious that the cases in which it is applicable must be very few.

Another plan† has been proposed to destroy calculi without having recourse to the formidable operation of lithotomy, and which we have detailed in a preceding part of the present Number. We allude to the decomposition of those bodies by means of the galvanic pile. Having referred to the experiments of MM. PREVOST and DUMAS, we have nothing to add, either *pro* or *con*, in the present stage of the inquiry: it is not yet far enough advanced to enable us to give a very favourable opinion of its ultimate adoption in the human subject.

We have to notice some cases of *burns,* recorded by Dr. CUMIN, of Glasgow, together with the dissections, and remarks arising out of them, and which appears to us to deserve attention. They are immediately followed by a fatal case of burn, and the dissection of the body, related by Mr. SWAN, and which tends, in a very satisfactory manner, to confirm the views entertained by the first-named gentleman. The treatment of these accidents has been for a long time in a very uncertain and unsettled state: the impression made by Mr. KENTISH's pamphlets has not

---

* *Journal Universel des Sciences Medicales,* Aout.

† *Archives génerales de Médecine,* Septembre.

yet ceased to influence many practitioners; and, though that gentleman's opinions have been combated,—and successfully, we conceive,—by several respectable writers, (Mr. Dickenson, in particular,) yet no regular or systematic mode of treatment seems to have been adopted by the profession generally. Our own opinion most decidedly is, that the phenomena of inflammation are alone to be dreaded after extensive accidents of this description; that all stimulating applications, whether external or internal, are consequently injurious; and that, after the stage of collapse has ceased, and re-action has decidedly commenced, our treatment must be strictly antiphlogistic, in proportion to the extent of the injury. Let us not consult theory to the exclusion of practical knowledge. The cooling and evaporating external applications to burns and scalds are those which produce immediate ease and comfort, and we shall not be easily persuaded that a removal of pain, and of pain so intense and violent as that which accompanies an extensive burn, can be otherwise than correct and judicious practice.

Dr. Cumin* has recorded five fatal cases of burns, four of which occurred in children. In the first case, the state of collapse continued for two days, and is described as accompanied with cold feet, a sense of languor and chilliness, great thirst, and a small weak pulse. On the third day, however, the face was flushed, pulse 128; and, the next day, the child complained, in addition, of great pain in her chest and back: in short, sloughs began to form on the burnt surface, and the thumb appeared completely sphacelated. It is necessary to observe, that the turpentine dressings were adopted in the first instance, and, after a lapse of some days, great relief, it is said, was experienced in changing these dressings for vinegar cloths. The child died exhausted in little more than a month. On dissection, marks of inflammatory action pervaded the whole lining of the chest. We cannot afford space to detail another of these cases, but they all prove the great degree of action induced by the accident, and the obvious tendency to inflammation in the internal cavities either of the brain, chest, or abdomen. In all, the turpentine dressings had been employed, and opium, wine, and cordials freely given.

Mr. Swan's case was also a fatal one, in a child five years of age, and who lived only two days after the accident. Oil was applied to the burn in the first instance; and, on his admission to the hospital, cerate of rosin, to which spirit of turpentine had been added. The post-mortem examination showed vascularity in the brain, and some black spots and stripes, like sloughs, in the villous coat of the stomach. We quote, in conclusion, the following judicious remarks of Mr. Swan:—"If any conclusion can be drawn from this case, respecting those who have been burnt, I think opiates ought not to be given, nor any stimulants, but that the patient should be purged; and, if coma comes on, and the head is much affected, leeches should be applied to the temples. There can be no doubt that the diseased appearances of the head and stomach were produced by the irritation of the skin; and, therefore, I think all applications tending to increase this state should be avoided."

---

* *Edinburgh Medical and Surgical Journal*, No. 76.

Having so fully discussed the works of Sir Astley Cooper and Mr. Earle, on *fractures of the neck of the thigh-bone,* we have no intention of renewing the subject; but we take this opportunity of reverting to Mr. Earle's book, for the purpose of noticing that portion of it which we did not, in our anxiety to do justice to the controversial part of the volume, even allude to;—we mean the *remarks on injuries in the vicinity of the shoulder-joint,* with the description of an apparatus for the more effectually securing the upper extremity. Every surgeon must be aware of the difficulty that presents itself in adjusting those fractures of the clavicle which occur towards the centre of that bone, and the inadequacy of the means commonly employed to preserve the fractured ends of the bone level, which, indeed, can only be done by keeping the whole limb in a perfectly passive state. Mr. Earle has turned his attention to a contrivance of this kind, which he has found equally applicable to other injuries occurring near the shoulder-joint,—such as fracture of the acromion, fracture of the neck of the scapula, or of the coracoid process. In all these accidents, our author observes, that the leading principle is to unite the whole upper extremity with the trunk, that they may form one body and move together; that it is not possible to restrain the motion of one part, whilst the other is left at liberty, in consequence of the totality of motion in the scapula, clavicle, and humerus. Acknowledging the correctness of the principles of Dessault's apparatus, which however he considers as imperfect, for reasons which appear to us sufficiently founded, Mr. Earle proposes the following apparatus:

"The apparatus consists of a strong sleeve, made of double jean or linen cloth, which reaches from half-way up the upper arm, is fitted to the elbow, when bent to an angle of about seventy-five degrees, and terminates, like the sleeve of a straight waistcoat, in a cul-de-sac. This is applied to the arm, and secured by straps, or a lace and eyelet holes: at the extremity of this sleeve a band of strong webbing is attached, which is passed round the body, and fixed to a broad buckle, which is fastened to a belt of calf-skin, lined with wash-leather, about three inches broad, which is passed round the injured arm, just below the insertion of the deltoid. The action of this sleeve and strap is to prevent any motion in the arm or forearm, and to bind it firmly to the trunk. To support the elbow in any position which may be required, I employ a leather cap, adapted to the extremity of the elbow, and hollowed out at its centre for the olecranon. This is put on over the sleeve, and from it two broad bands of webbing pass obliquely up to the opposite shoulder; one in front of the thorax, and the other behind. These bands are affixed to two broad buckles, which are attached to a leather shoulder-cap, made of calf-skin, well padded and lined with wash-leather, which is adapted nicely to the shoulder by means of a buckle and strap, which passes under the axilla. By tightening or slackening these bands, the elbow may be either confined close to the side or brought forward, as in the position required for fractures of the clavicle or the coracoid process; and it may be permanently and steadily fixed in that position. Another strap may be brought down from the anterior oblique strap, and passed round the wrist, to assist in supporting the weight of the extremity.

"The advantages proposed in this apparatus are, greater security to the fractured limb, and greater comfort to the patient. The drawing from the point of the elbow to the opposite shoulder, and fixing the bands to buckles, gets rid of the galling of bandages across the root of the neck and over the shoulder; which, in persons with irritable skins, so constantly follows the application of rollers: whilst, at the same time, it is much more secure and less likely to slip than any bandages. The sleeve being rather loose, allows of some motion in the fingers and forearm; whilst the band, which passes round the body, and is fixed to the belt just below the axilla, in the affected arm, most effectually restrains all motion in the arm and shoulder, and keeps back the upper part of the humerus. There being no circular bands round the chest, the objection said to arise from the use of Desault's plan, in females and persons with difficult respiration, are obviated; for the band, which passes obliquely across the front of the chest, may always be made to pass between the mammæ without pressing on either. Another advantage proposed by this apparatus is the opportunity afforded of observing the affected shoulder, and making any applications which may be deemed requisite, without in the least deranging the apparatus. In fractures of the clavicle, it will be right to employ a pad, or firm cushion, under the axilla, for the purpose of throwing out the shoulder, in the same manner as proposed by Desault. So, likewise, in fractures of the acromion, a cushion may be interposed between the inner condyle of the humerus and the chest, to throw out the elbow and relax the deltoid: these modifications will not interfere with the simplicity of the apparatus, and are what a judicious practitioner would employ under any circumstances. When this apparatus is properly adapted, the whole limb is so firmly and securely fixed, that the patient may be allowed to rise from his bed, and follow his usual avocations, after a few days have elapsed, and any inflammatory symptoms which may have presented themselves have been allayed."

It is proper to state, that our author has not yet had an opportunity of trying this bandage in a case of this description.

It was our intention to have made some remarks on *diseases of the spine*, and the different modes of treatment recommended for their cure; but we have postponed our remarks in consequence of the very recent publication of Mr. Shaw's work; an analysis of which will be found in the present Number. We likewise refer our readers, with pleasure, to the last part of Mr. Bampfield's paper on *curvatures of the spine*, contained in one of the early Numbers of the last volume of our Journal.

Our catalogue of surgical books during the last half year is not extensive, but to say any thing that has not been said before becomes every day a more difficult task: we must, however, notice a pamphlet of Mr. Green's, on the *fumigating bath*; a work in which the reader will find the rise and progress of these establishments succinctly detailed, with a candid and impartial account of the benefit likely to be derived from the employment of this remedy in various diseases, particularly those of the skin, and in which we have ourselves seen their great efficacy strikingly displayed.

Among the continental surgical works, we have to notice M. Delpech's volume of *Clinical Surgery*, Mr. Boyer's eighth volume of *Maladies Chirurgicales*, both excellent works, and M. Begin's application of the *physiological doctrines to Surgery*. This latter work, we confess, perhaps to our shame, does not strike us as possessing much merit, nor indeed can we discover scarcely any novelty. There are few facts or principles of any importance contained in this work, but what are familiarly known to surgeons on this side of the Channel. We need scarcely be told that inflammation is the great pivot upon which most surgical diseases turn; and that to excite, or to repress it, is one of the most common objects of the surgeon's attention.

We must refer our readers to the Medical Repository for October, (page 318,) for a description of an *improved trephine*, of which we cannot venture to give an account without the assistance of a plate; and we observe that Mr. Weiss has invented an *improved speculum*, or *dilator ani*, the principle of which is extremely simple, and which, in cases of tumors, either in the vagina or rectum, will certainly very much facilitate their examination and extirpation, by affording a good view of the diseased part.

We had prepared notes relative to the other branches of medical science, but, on re-perusing them, we cannot find any thing of sufficient importance to justify us in trespassing further on the other departments of the Journal.

